# Supplementary material for: Aggregation promotes charge separation in fullerene-indacenodithiophene dyad
Source: Nat Commun. 2024 Jul 6;15:5681. doi: 10.1038/s41467-024-50001-z (PMC11227505; doi:10.1038/s41467-024-50001-z)
Supplement: Supplementary file 1 — Supplementary Information [file 41467_2024_50001_MOESM1_ESM.pdf]

Supplementary Information

Aggregation Promotes Charge Separation in Fullerene-Indacenodithiophene Dyad

Chong Wang[1,2], Bo Wu[1,2]*, Yang Li[3], Shen Zhou[4], Conghui Wu[5], Tianyang Dong[1], Ying Jiang[1,2], Zihui Hua[1,2], Yupeng Song[1,6], Wei Wen[1,7], Jianxin Tian[1,2], Yongqiang Chai[8], Rui Wen[1,2] and Chunru Wang[1,2]*

[1] Beijing National Laboratory for Molecular Sciences, Key Laboratory of Molecular Nanostructure and Nanotechnology, Institute of Chemistry, Chinese Academy of Sciences, Beijing 100190, China

[2] University of Chinese Academy of Sciences, Beijing 100049, China

[3] School of Science, Beijing University of Posts and Telecommunications (BUPT), Beijing 100876, China

[4] College of Science, Hunan Key Laboratory of Mechanism and Technology of Quantum Information, National University of Defense Technology, Changsha 410003, China

[5] Spin-X Institute, School of Chemistry and Chemical Engineering, South China University of Technology, Guangzhou 511442, China

[6] Key Laboratory of Photochemical Conversion and Optoelectronic Materials and CityU-CAS Joint Laboratory of Functional Materials and Devices, Technical Institute of Physics and Chemistry, Chinese Academy of Sciences, Beijing 100190, China

[7] Beijing National Laboratory for Molecular Sciences, CAS Key Laboratory of Organic Solids, Institute of Chemistry, Chinese Academy of Sciences, Beijing 100190, China

[8] Department of Chemistry and Pharmacy and Interdisciplinary Center for Molecular Materials (ICMM), Friedrich-Alexander-Universität Erlangen-Nürnberg, Erlangen 91058, Germany

Contents

Supplementary Note 1: Synthesis and characterizations 3

Supplementary Note 2: HPLC separation and purification 4

Supplementary Note 3: ^{1}H NMR 5

Supplementary Note 4: MALDI-TOF MS 6

Supplementary Note 5: Morphology characterization of thin-film 6

Supplementary Note 6: Steady-state absorption and emission spectra 7

Supplementary Note 7: Electrochemistry 11

Supplementary Note 8: Transient absorption spectra 14

Supplementary Note 9: Pulsed electron paramagnetic resonance 37

Supplementary Note 10: Computational studies 38

Supplementary Note 11: Photoelectronic switch 43

Supplementary Note 12: Photocurrent response 44

Supplementary Reference 46

Supplementary Note 1: Synthesis and characterizations

Three indacenodithiophene precursors are purchased from *Organtec Ltd*. The fullerene-indacenodithiophene D-A covalent small molecules (IT-C_{60}) are synthesized by *prato* reaction[1]. All reactions are performed in nitrogen, and details depict as follows (**Supplementary Fig. 1**):

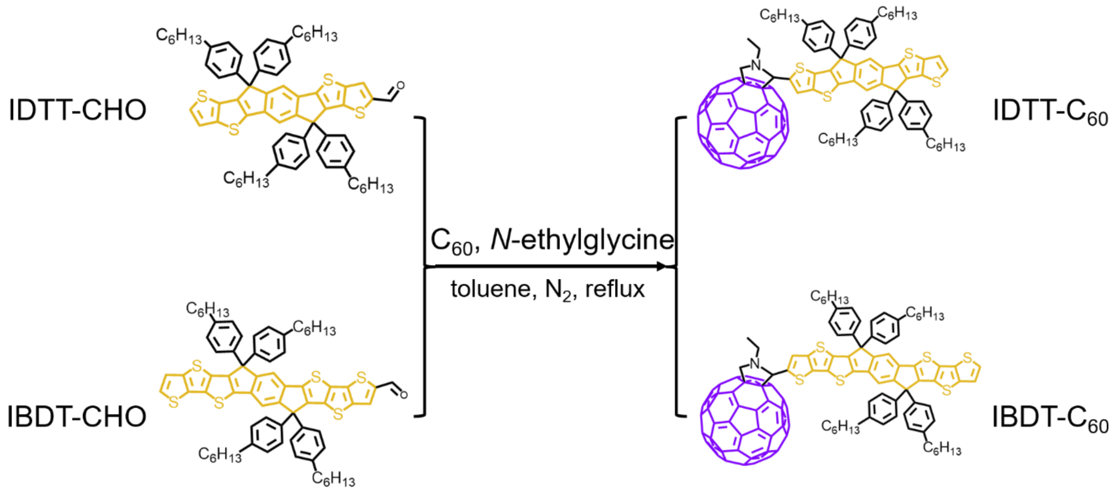

Supplementary Fig. 1 Synthetic methodologies. Synthesis routes of ID (IDTT-C_{60}) and IB (IBDT-C_{60}) via the prato reaction.

***N*-Ethyl-2-indacenodithieno[3,2-b]thiophene-[6,6]-*I_h*-C_{60}-fulleropyrrolidine** (IDTT-C_{60}, ID, $C_{132}H_{81}NS_4$). 508.22 mg IDTT-CHO (0.49 mmol, 1 equiv), 488.66 mg C_{60} (0.68 mmol, 1.4 equiv) and 45.66 mg *N*-ethylglycine (0.44 mmol, 0.9 equiv) were dissolved in 250 mL toluene. The reactants mixture was heated and refluxed at 130 °C for 6 h under N_2 protection. When the reaction completed, the solvent was evaporated, and the crude product was separated by silica gel column chromatography (eluent; petroleum ether and toluene 3:1) and further purified by HPLC with buckprep column (toluene, 6 mL/min). ^{1}H NMR ($CDCl_3$, 400 MHz, δ/ppm): δ 7.47(s, 2H), δ 7.11-7.17(m, 8H), δ 7.01-7.08(m, 8H), δ 6.95(d, 2H), δ 5.46(d, 1H), δ 5.14(s, 1H), δ 4.22(d, 1H), δ 3.65(d,1H), δ 2.52-2.56(m, 8H), δ 1.43-1.53(m, 10H), δ 1.18-1.33(m, 27H), δ 0.84-0.89(m, 12H). MALDI-TOF MS (m/z): 1808.531 (M^+).

***N*-Ethyl-2-indacenobis(dithieno[3,2-b;2',3'-d]thiophene-[6,6]-*I_h*-C_{60}-fulleropyrrolidine** (IBDT-C_{60}, IB, $C_{136}H_{81}NS_6$). 150.45 mg IBDT-CHO (0.13 mmol, 1 equiv), 140.01 mg C_{60} (0.194 mmol, 1.5 equiv) and 12.22 mg *N*-ethylglycine (0.119 mmol, 0.9 equiv) were dissolved in 120 mL toluene. Under N_2 protection, the mixture was refluxed at 130 ^oC for 6 h. After the reaction completed, the solvent was evaporated, and the crude product was subjected to the silica gel column chromatography (eluent; petroleum ether and toluene 3:1) and further purified by HPLC with buckprep column (toluene, 6 mL/min). ^1H NMR ($CDCl_3$, 400 MHz, δ/ppm): δ 7.50-7.52(m, 2H), δ 7.3(d, 2H), δ 7.19-7.22(m, 8H), δ 7.06-7.1(m, 8H), δ 5.46(s, 1H), δ 5.14(d, 1H), δ 4.20(d, 1H), δ 3.54(d,1H), δ 2.50-2.58(m, 8H), δ 1.52-1.61(m, 10H), δ 1.23-1.35(m, 27H), δ 0.82-0.88(m, 12H). MALDI-TOF MS (m/z): 1920.459 (M^+).

Supplementary Note 2: HPLC separation and purification

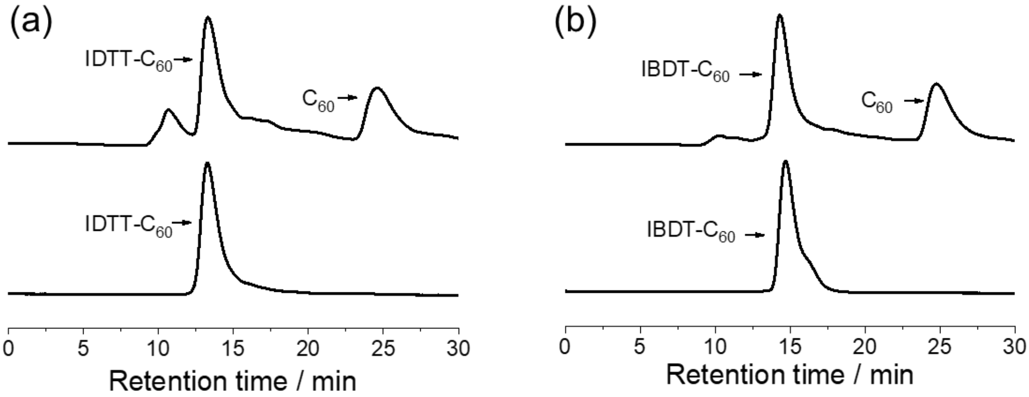

Supplementary Fig. 2 HPLC separation and purification. (a) HPLC profiles of raw reaction mixture (top) and purified monoadduct after separation (bottom) of ID. (b) HPLC profiles of raw reaction mixture (top) and purified monoadduct after separation (bottom) of IB. Buckprep column: 10 × 250 mm; eluent: toluene, 6 mL / min.

Supplementary Note 3: ^{1}H NMR

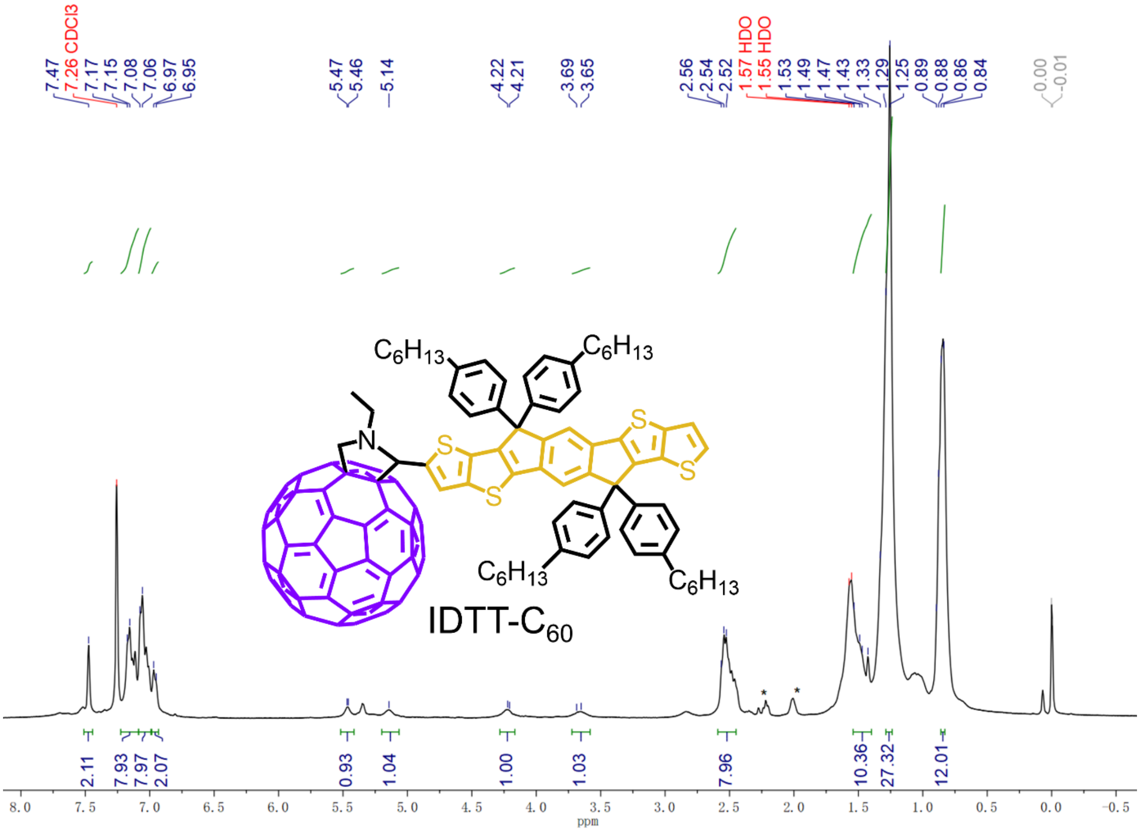

Supplementary Fig. 3 ^{1}H NMR characterization. ^{1}H NMR spectrum of ID in $CDCl_3$.

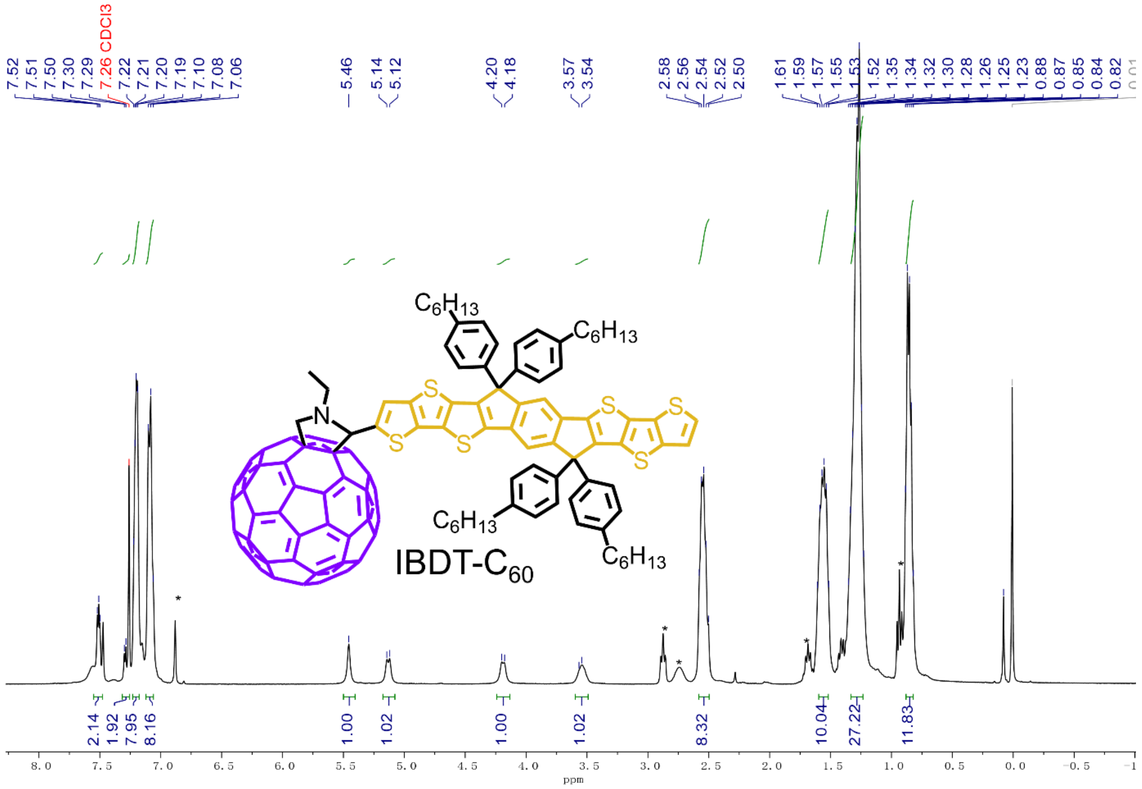

Supplementary Fig. 4 ¹H NMR characterization. ¹H NMR spectrum of IB in $CDCl_3$.

Supplementary Note 4: MALDI-TOF MS

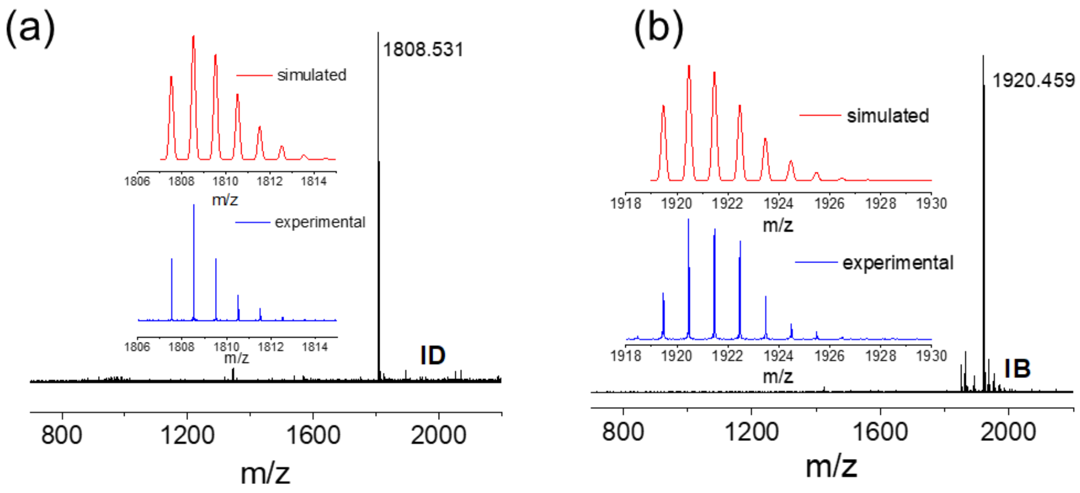

Supplementary Fig. 5 MALDI-TOF mass spectra characterization. (a) MALDI-TOF MS of ID. (b) MALDI-TOF MS of IB. The insets show the simulated (top) and experimental (bottom) isotope distributions.

Supplementary Note 5: Morphology characterization of thin-film

To gather insight into the morphology of the film, we have incorporated characterizations including atomic force microscope (AFM), and transmission electron microscope (TEM). The height and phase images obtained from AFM reflect a smooth and uniform surface of the films, with a root-mean-square (RMS) roughness less than 0.5 nm, indicating a homogeneous structure. TEM provides additional evidence for the homogeneity of films (**Supplementary Fig. 6**)[2-4].

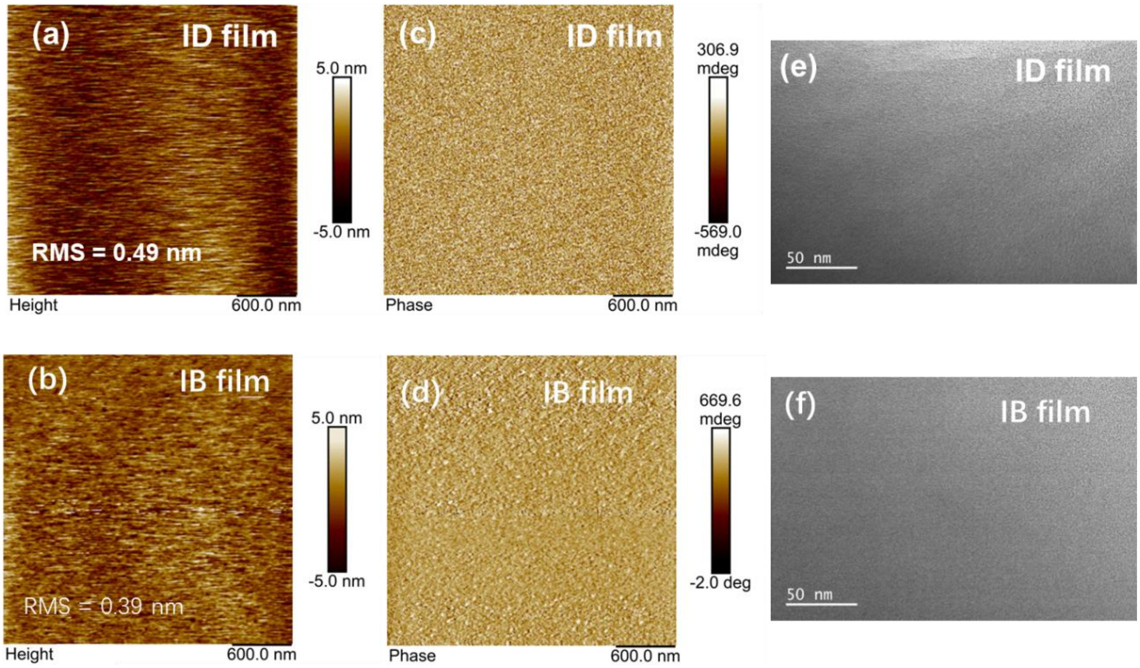

Supplementary Fig. 6 Morphology characterization. Height images of ID (a) and IB (b), and phase images of ID (c) and IB (d) obtained from AFM. Scale bar: 600 nm. TEM images of ID (e) and IB (f). Scale bar: 50 nm.

Supplementary Note 6: Steady-state absorption and emission spectra

We added the steady-state absorption and emission spectra of IB in *o*-DCB, aggregated film, and PMMA-diluted film, along with the precursor IBDT, as shown in **Supplementary Fig. 7**. These results are similar to ID, as discussed in the main text.

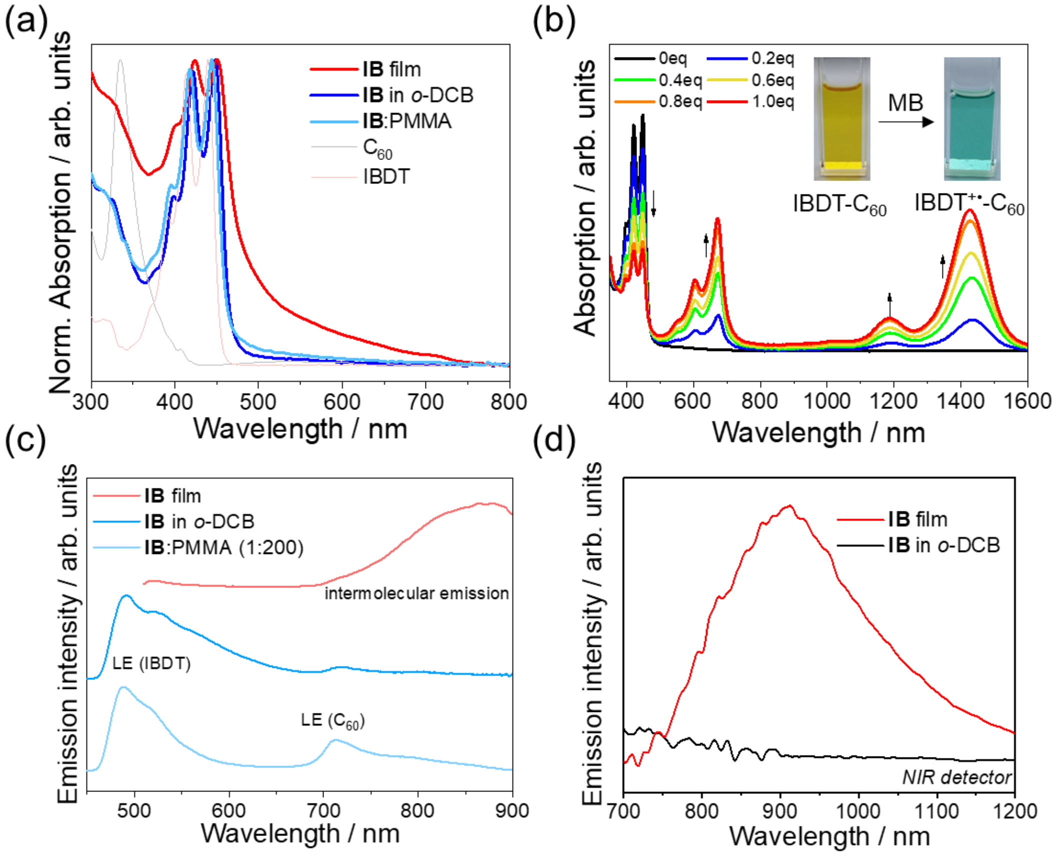

Supplementary Fig. 7 Steady-state spectra of IB. (a) Absorption spectra of IB in *o*-DCB and film, PMMA-diluted IB film (IB:PMMA=1:200, w:w) and the precursors IBDT and C_{60} in *o*-DCB. (b) Change in the spectra upon chemical oxidation under the addition of 0 to 1 equivalent (equiv) "Magic Blue" (MB) in *o*-DCB for IB; inset: photographs of the color change before (left) and after (right) chemical oxidation. (c) Photoluminescence spectra of IB film, IB in *o*-DCB, and PMMA-diluted IB film, excited at 400 nm and recorded by visible detector. (d) Photoluminescence spectra of IB in *o*-DCB and film recorded by NIR detector.

The energy of the localized excited state (LES) of IDTT or IBDT can be estimated by the normalized absorption and emission spectra, using the intersection wavelength (**Supplementary Fig. 8**)[5].

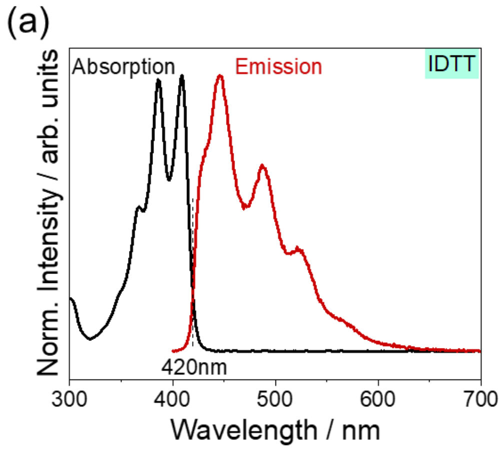

Supplementary Fig. 8 Calculation of the localized excited state energy. Normalized absorption and emission spectra of (a) IDTT and (b) IBDT. The wavelength (λ) at the intersection of the normalized absorption and emission spectra can be used to estimate the 0-0 transition energy (1240/λ, eV), representing the energy of the localized excited state (LES) of IDTT or IBDT.

To illustrate the negligible intermolecular interaction in solution, we measured the concentration dependent absorption spectra in *o*-DCB using 1 mm optical path quartz cell (**Supplementary Fig. 9**). With the increase of concentration, the spectra do not show obvious redshift, and the absorbance has a good linear relationship with concentration, indicating that no intermolecular aggregation exists in solution. The sample concentration we used for transient absorption measurement, 10^{-4} M, is included in the linear range.

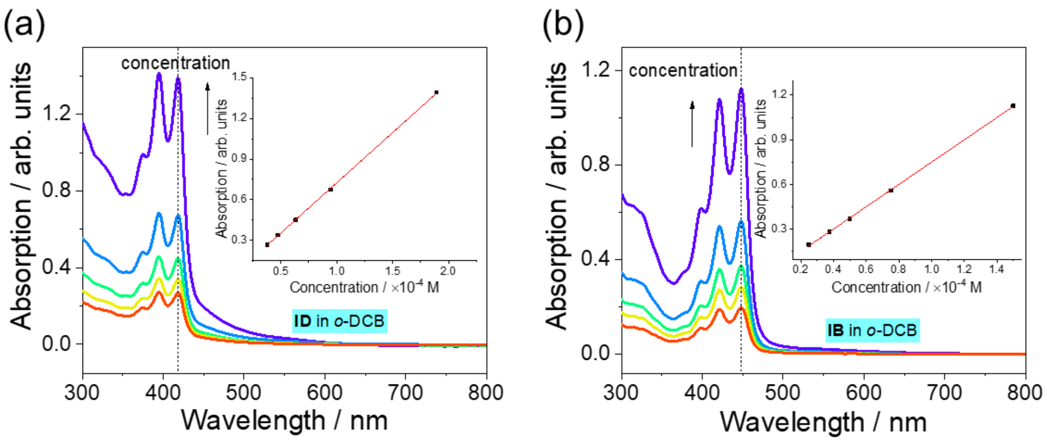

Supplementary Fig. 9 Concentration dependent absorption spectra in *o*-DCB. (a) Absorption spectra of ID under different concentration. (b) Absorption spectra of IB under different concentration. Inset: linear dependence of absorbance on concentration, recorded at 420 nm for ID and 450 nm for IB.

In addition, the absorption and emission spectra of the blend systems are shown in **Supplementary Fig. 10**.

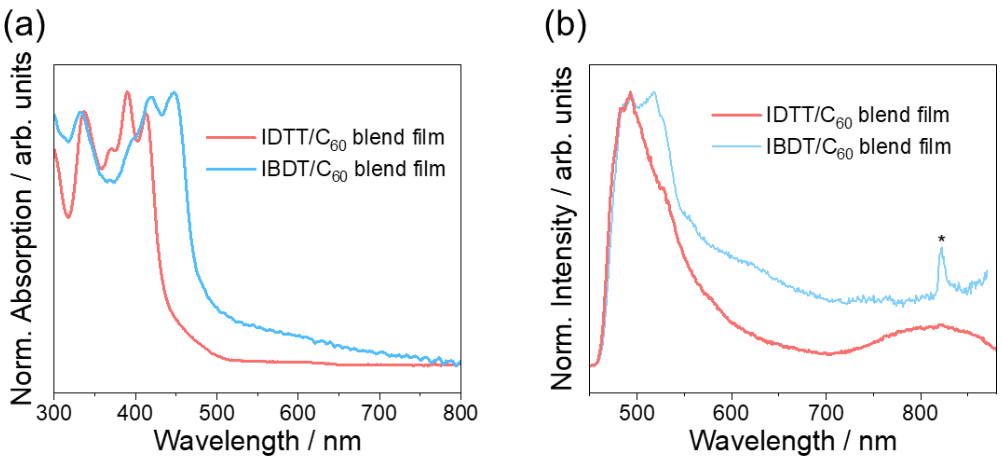

Supplementary Fig. 10 Steady-state absorption and emission spectra of blend films. (a) Absorption spectra of IDTT/C_{60} and IBDT/C_{60} blend films. (b) Emission spectra (excited at 400 nm) for IDTT/C_{60} blend and IBDT/C_{60} blend films.

The photoluminescence (PL) lifetime of the thin-films was measured using time-correlated single-photon counting (TCSPC). Due to the weak PL signal of the sample and the strong background signal from the near-infrared detector, it was challenging to distinguish the PL lifetime around the emission center, 900 nm. As a result, we utilized a visible detector to investigate the luminescent decay process at 820 nm (falling within the PL range), as shown in **Supplementary Fig. 11**. The lifetimes are estimated to be about 5.2 ns for ID film, and 5.6 ns for IB film.

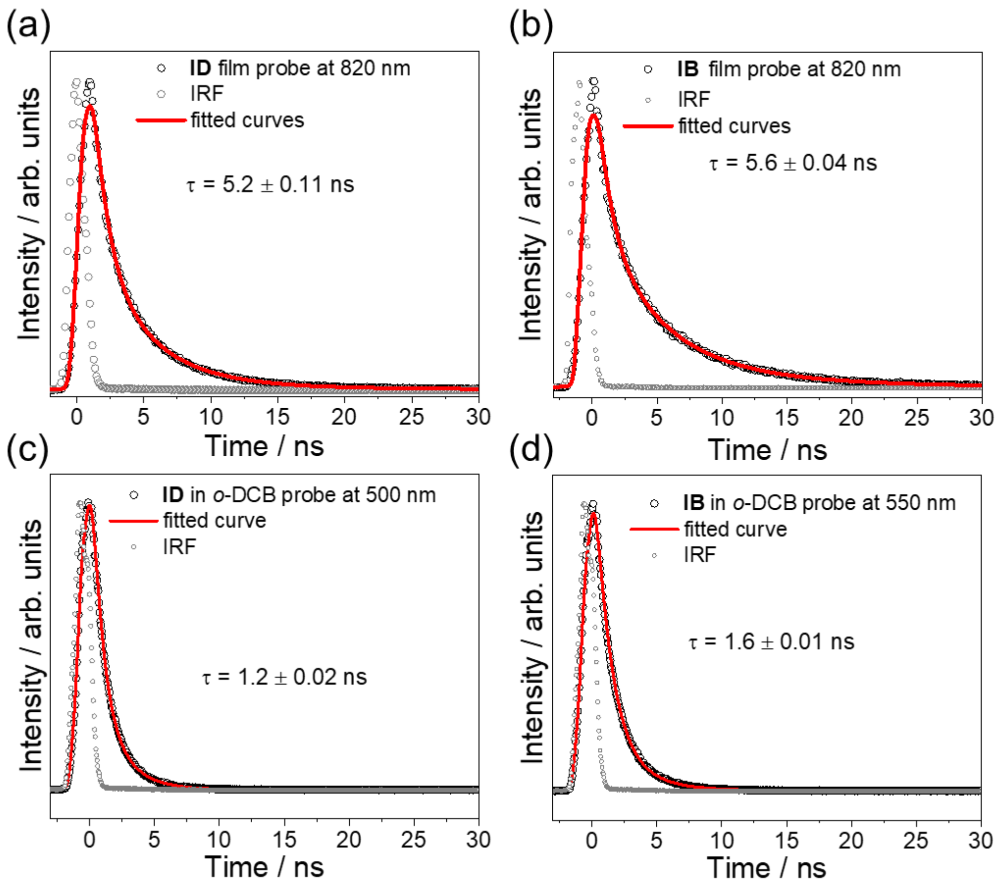

Supplementary Fig. 11 Photoluminescent lifetime measurement. (a, b) Photoluminescent kinetics of ID and IB film, recorded at 820 nm and excited at 405 nm. (c, d) Photoluminescent kinetics of ID (recorded at 500 nm) and IB (recorded at 550 nm) in *o*-DCB excited at 405 nm.

Supplementary Note 7: Electrochemistry

Differential pulse voltammetry (DPV) studies were carried out to study the electrochemical properties of IT-C_{60} and precursors. As shown in **Supplementary Figs. 12** and **13**, the first oxidation potential ($^{1}E_{ox}$) of IBDT (0.23 V *vs.* Fc/Fc^{+}) is lower than IDTT (0.32 V *vs.* Fc/Fc^{+}), indicating the stronger electron-donating ability of IBDT donor. We note that the difference between the electrochemical properties of ID and IB decreased after aggregation, as well as the changed electrochemical bandgap, which is probably to be influenced by the intermolecular interaction after aggregation.

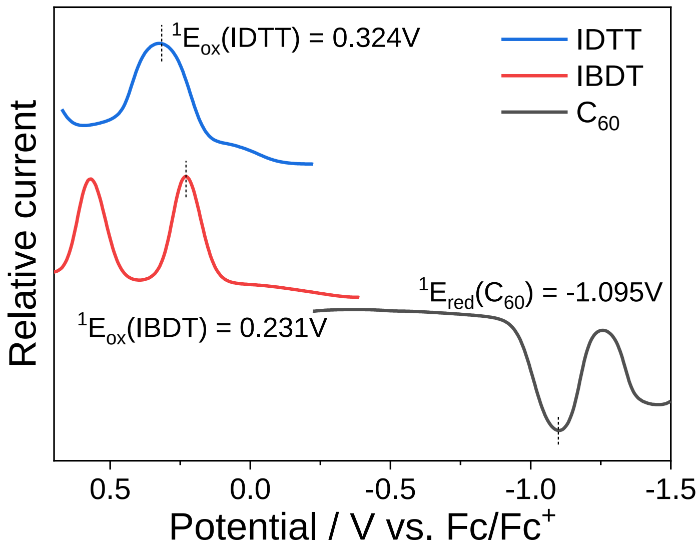

Supplementary Fig. 12 Differential pulse voltammograms (DPV) measurement. DPV of the precursor donor IDTT, IBDT and acceptor C_{60} were recorded in *o*-DCB containing 0.05 M $TBAPF_6$. The ferrocene/ferrocenium (Fc/Fc^+) was used as the internal standard reference.

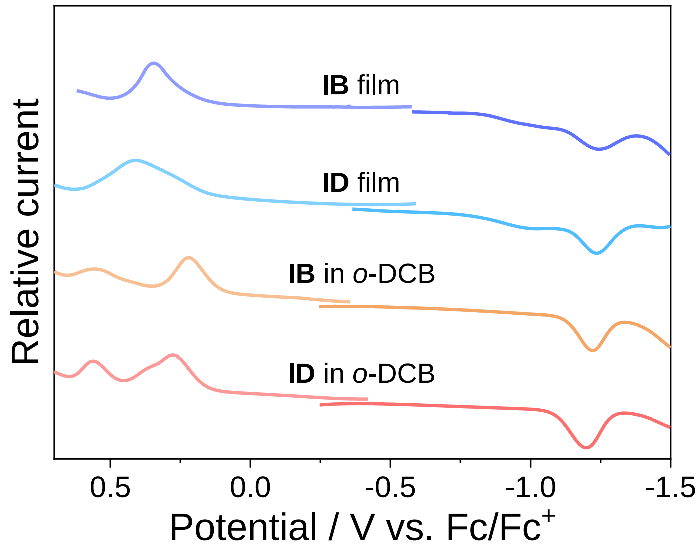

Supplementary Fig. 13 Differential pulse voltammograms (DPV) measurement. Differential pulse voltammograms (DPV) of ID and IB in solution (containing 0.05 M $TBAPF_6$ in deaerated *o*-DCB) and film (containing 0.05 M $TBAPF_6$ in deaerated acetonitrile). The ferrocene/ferrocenium (Fc/Fc^+) was used as the internal standard reference.

The Gibbs free energies of charge separation (ΔG_{CS}) and recombination (ΔG_{CR}), that is, driving force in *o*-DCB can be estimated using the Rehm-Weller equation (Supplementary Equation (1))[6]:

$$\begin{aligned}\Delta G_{CS} &= e\left({}^{1}E_{ox}(D) - {}^{1}E_{red}(A)\right) - E_{0,0} + C + S \\ \Delta G_{CR} &= -\Delta G_{CS} - E_{0,0}\end{aligned} \tag{1}$$

where ${}^{1}E_{ox}(D)$ and ${}^{1}E_{red}(A)$ are the first oxidation and reduction potential of studied IT (D) and C_{60} (A); $E_{0,0}$ is the zero-zero transition energy, which is 1.76 eV for the singlet excited state of C_{60}[7]; *C* and *S* are related to solvents and molecular structure (Supplementary Equation (2)):

$$C = -\frac{e^2}{4\pi\varepsilon_0\varepsilon_s d_{DA}}, \quad S = -\frac{e^2}{4\pi\varepsilon_0}\left(\frac{1}{2r_D} + \frac{1}{2r_A}\right)\left(\frac{1}{\varepsilon_{ref}} - \frac{1}{\varepsilon_s}\right) \tag{2}$$

r_D/r_A are radius of donor/acceptor; d_{DA} is the central distance between donor and acceptor in D-A dyads; ε_s and ε_{ref} are the dielectric constants of the given solvent and the reference solvent used in electrochemistry, respectively (*o*-DCB: ε=9.93).

Herein, the ${}^{1}E_{red}$ of C_{60} and ${}^{1}E_{ox}$ of IDTT and IBDT are used to estimate the driving force of CS and CR. Combining with the geometric optimization according to DFT calculation at B3LYP-D3(BJ)/6-31G* level, the Gibbs free energy of CS and CR for ID and IB can be summarized in **Supplementary Table 1**. Clearly, the CS driving force of IB is larger than that of ID, while the CR driving force is smaller. Besides, the higher dipolar PhCN provides larger CS driving force and smaller CR driving force than *o*-DCB. These are consistent with the CS kinetics obtained from transient absorption.

Supplementary Table 1. Electrochemical properties. Summary of the electrochemical properties of ID and IB, as well as the Gibbs free energies (ΔG) during charge separation (CS) and charge recombination (CR).

Molecule	$^1E_{ox}$ / V	$^1E_{red}$ / V	*o*-DCB		PhCN	
			ΔG_{CS} / eV	ΔG_{CR} / eV	ΔG_{CS} / eV	ΔG_{CR} / eV
ID	0.32	-1.09	-0.57	-1.19	-0.83	-0.93
IB	0.23	-1.09	-0.65	-1.11	-0.90	-0.86

On the basis of the steady-state spectra and electrochemical analysis, **Supplementary Table 2** summarizes the excited-state energy levels, including localized excited states (LES) for the donor and acceptor, charge-separated states (CSS) in solution and films, as well as intermolecular CSS in films (i.e., free carriers, FCs), which will be involved in the subsequent analysis of excited-state photophysical properties[8].

Supplementary Table 2. Summary of the excited states energy levels. Calculated energy level of localized excited state (LES), charge-separated state (CSS), and free carriers (FCs) for ID, IB and the precursors in *o*-DCB, PhCN or film.

Energy level / eV	LES donor	LES acceptor	CSS in *o*-DCB	CSS in PhCN	CSS film	FCs film
ID	2.95	1.76	1.19	0.93	1.41	1.42
IB	2.78	1.76	1.11	0.86	1.32	1.36

Supplementary Note 8: Transient absorption spectra

To distinguish the localized excited states (LES) characteristics, transient absorption spectra (TAS) of two IT-CHO precursors are investigated in *o*-DCB, wherein, upon 410 nm excitation, LES are firstly generated. As shown in **Supplementary Fig. 14**, for IBDT-CHO, the excited state absorption (ESA) peak of the LES locates around 780 nm, and significant negative ground state bleaching (GSB) and stimulated emission (SE) signals are observed around 480 nm and 580 nm, respectively. For IDTT-CHO, similar

results are described, LES around 720 nm, GSB at 440 nm and SE at 520 nm. We also note some spectra evolution, such as SE redshift, LES blueshift, and increased absorption at NIR region within the first 50 ps. These probably result from the structural and solvent relaxation, which is not evident in IBDT-CHO because of the longer and more rigidity conjugate structure[9]. In addition, LES gradually appears in the long-wavelength region with the increase conjugation of fused-ring, reflecting that following the increasing conjugation, the excitons become more delocalized[10].

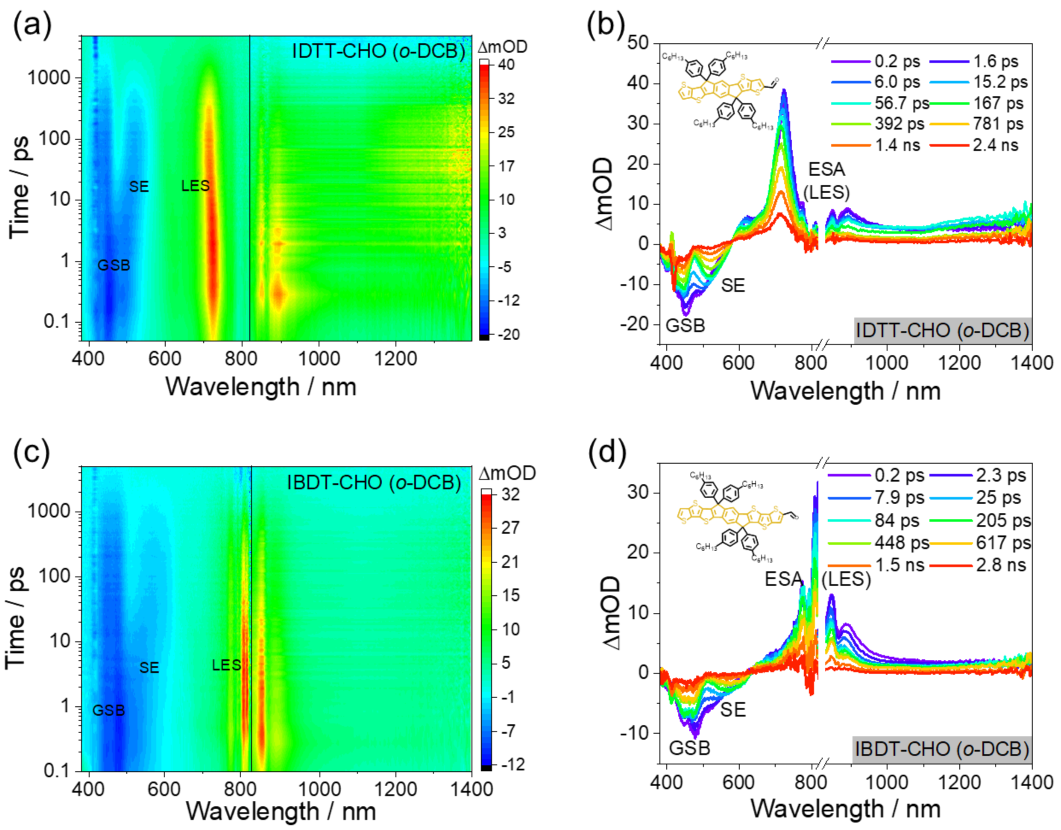

Supplementary Fig. 14 Femtosecond transient absorption of IT-CHO. (a and c) Contour plots of IDTT-CHO and IBDT-CHO in *o*-DCB following excitation at 410 nm. (b and d) Selected TA spectra of IDTT-CHO and IBDT-CHO in *o*-DCB following excitation at 410 nm.

We note that $C_{60}^{-\bullet}$ characteristic absorption is around 1010 nm[11], while, according to the chemical oxidation absorption spectrum, $IT^{+\bullet}$ also have strong absorption in this range. Thus, following charge separation, the ESA in this wavelength range appears as

a superposition of $C_{60}^{-\bullet}$ and $IT^{+\bullet}$. **Supplementary Fig. 15** describes the normalized the chemical oxidation absorption spectra, as well as the transient absorption spectra (TAS) after charge separation in the NIR region. It can be seen that the proportion of the absorption intensity of TAS near 1000 nm–1050 nm increases obviously, and the peak position performs slight blueshift relative to the chemical oxidation results. This probably reflects the $C_{60}^{-\bullet}$ absorption after excitation.

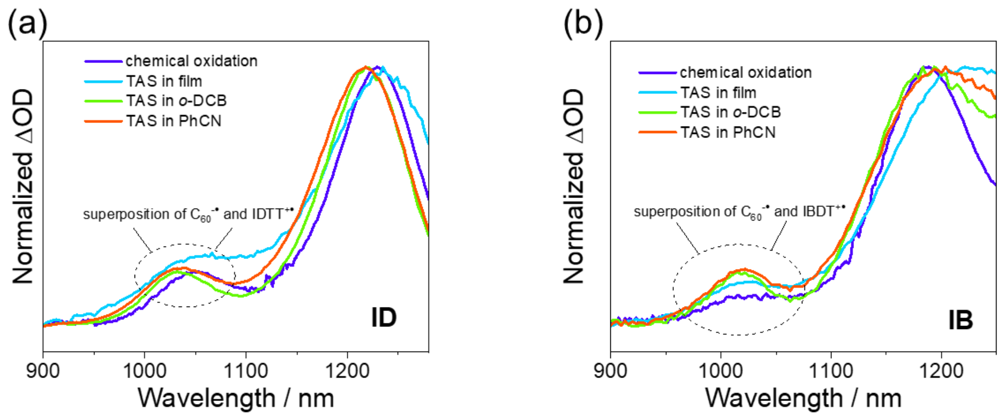

Supplementary Fig. 15 C_{60} anion absorption in the NIR region. (a) Comparison of the normalized chemical oxidation spectra and transient absorption spectra for ID. (b) Comparison of the normalized chemical oxidation spectra and transient absorption spectra for IB. The absorption of cations, 1230 nm for ID and 1200 nm for IB, were selected for normalization.

To clearly illustrate the excited-state evolution of ID in *o*-DCB under 410 nm excitation, we have divided the transient absorption spectral evolution into three stages, namely energy transfer, charge separation, and charge recombination processes. (1) Within the initial 1 ps, the excited-state absorption (ESA) of donor's LES, as well as the GSB and SE rapidly decay; but hole absorption at 620 nm has not yet appeared, indicating this stage belongs to the energy transfer process from donor to acceptor. Energy transfer quenches the GSB and SE of the donor, and simultaneously generates a weak ESA signal at 440 nm, which can be first observed when selectively exciting C_{60} at 350 nm (**Supplementary Figs. 16** and **17**), confirming the localized excited state of C_{60}. The excitation state absorption around 440 nm is likely the result of spectral

overlap. (2) Over the next 100 ps, the LES of C_{60} around 440 nm gradually decay, and at the same time, the hole absorption peak at 620 nm gradually emerges, indicating the charge separation process. The newly generated holes are distributed on the donor, leading to the regeneration of the donor's GSB. (3) Finally, the separated charges recombine to the ground state within 1 ns. The similar charge separation kinetics under excitation at 410 or 350 nm, suggesting that charge separation from the low-energy LES of C_{60} acts as the rate-determining step in solution (**Supplementary Fig. 18**).

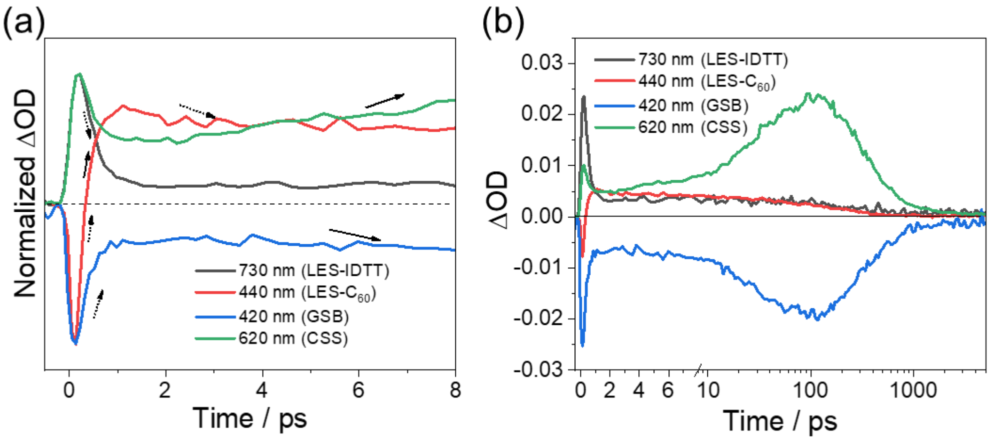

Supplementary Fig. 16 Excited state kinetic analysis. Depicting the excited-state kinetics associated with energy transfer (a), charge separation and charge recombination (b) of ID in *o*-DCB under 410 nm excitation; dashed arrows denote the decay of excited states, while solid arrows signify the generation of excited states in (a).

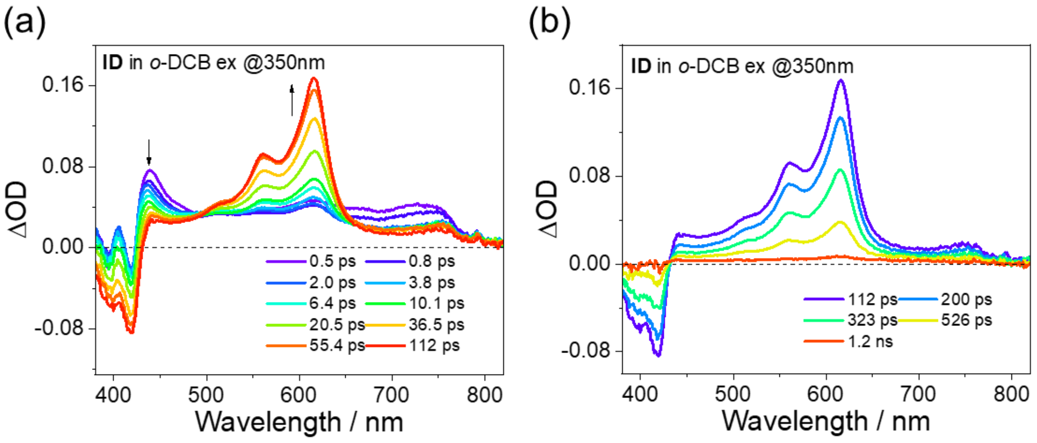

Supplementary Fig. 17 Transient absorption spectra (TAS) under 350 nm excitation. (a) TAS for ID in *o*-DCB under 350 nm excitation between 0.5 ps and 112 ps. (b) TAS for ID in *o*-DCB under 350 nm excitation between 112 ps and 1.2 ns.

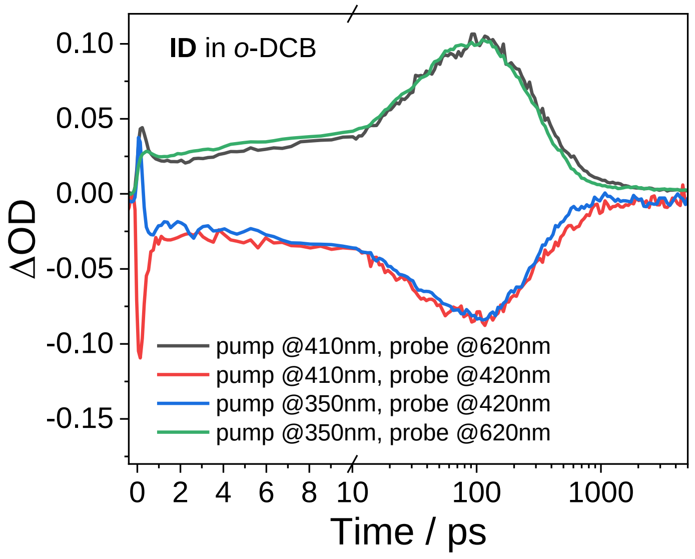

Supplementary Fig. 18 Comparison of the excited-state kinetics. Charge separation kinetics comparison under 410 nm and 350 nm excitation for ID in *o*-DCB.

The excited-state properties of IB are similar with ID. Upon excitation at 410 nm in *o*-DCB, the excited-state absorption of LES for IBDT at 780 nm, generating within 0.1 ps, and CSS at 680 nm forming within 50 ps, can be clearly distinguished (**Supplementary Figs. 19-21**). Detailed excited-state evolution processes also manifest by the energy transfer, charge separation and charge recombination (**Supplementary Figs. 22-24**). Different with ID, the charge separation and recombination time taken for IB in *o*-DCB is shorter than that of ID, due to the larger electron transfer driving force. The fitted time is 24.8 ps for CS, and 119.0 ps for CR.

The IB film also exhibits fast charge separation and slow charge recombination, compared to solution. The fitted time for IB film is about 0.7 ps for CS, according to femto-second transient absorption, and 25.8 μs for CR obtained by nanosecond transient absorption.

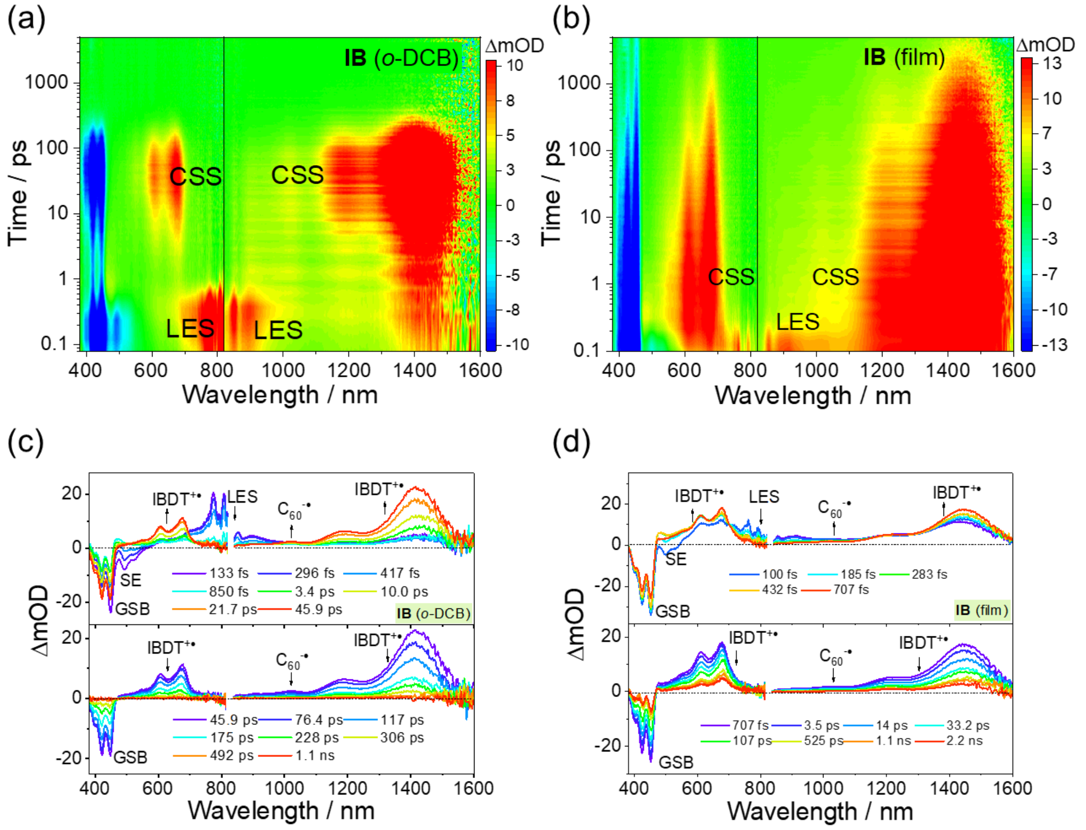

Supplementary Fig. 19 Femtosecond transient absorption results of IB. (a, b) Contour plots in *o*-DCB and film following excitation at 410 nm. (c, d) Selected TA spectra for IB in *o*-DCB and film.

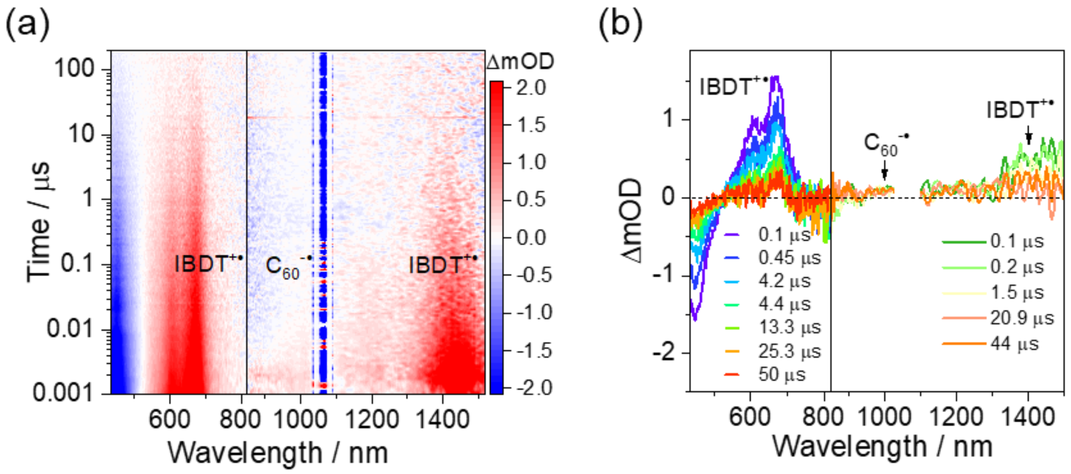

Supplementary Fig. 20 Nanosecond transient absorption results of IB film. (a) Contour plots following excitation at 410 nm. (b) Selected TA spectra.

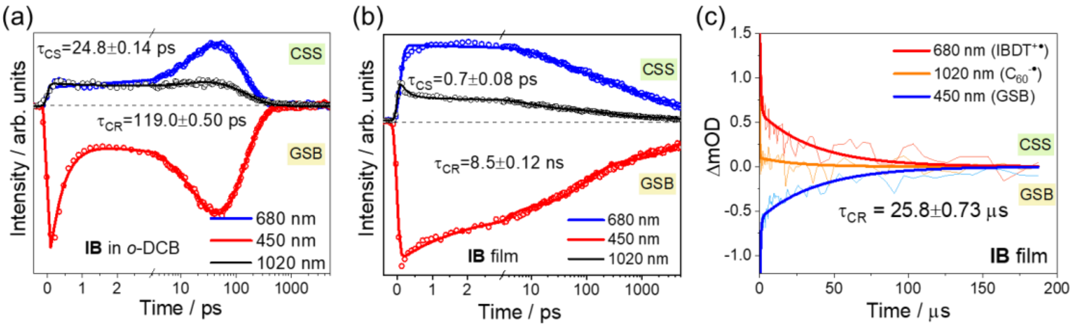

Supplementary Fig. 21 Time-absorption profile at CSS (680 nm for IBDT cation, and 1020 nm for C_{60} anion) and GSB (450 nm), as well as fitted curves for IB in (a) *o*-DCB, (b) film obtained from femtosecond transient absorption, and (c) in film obtained from nanosecond transient absorption.

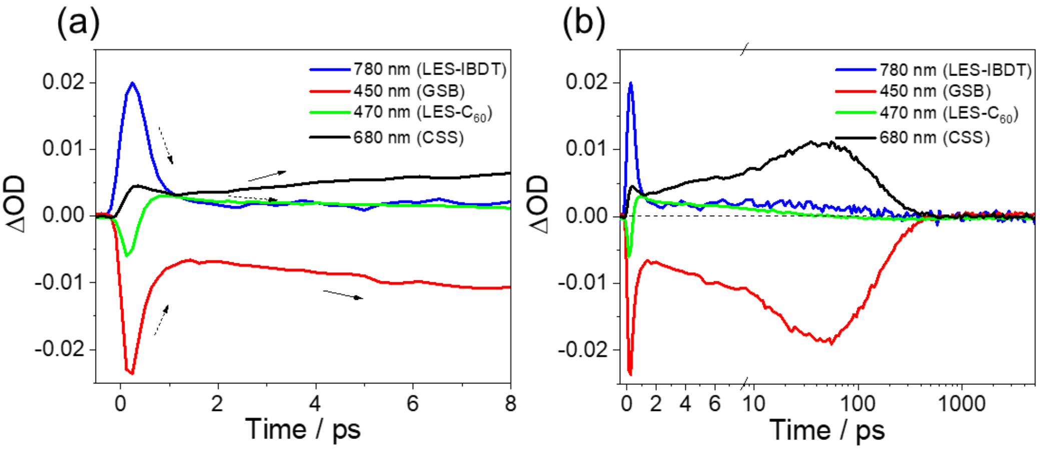

Supplementary Fig. 22 Excited state kinetic analysis. Depicting the excited-state kinetics associated with (a) energy transfer, (b) charge separation and charge recombination of IB in *o*-DCB under 410 nm excitation. Dashed arrows denote the decay of excited states, while solid arrows signify the generation of excited states in (a).

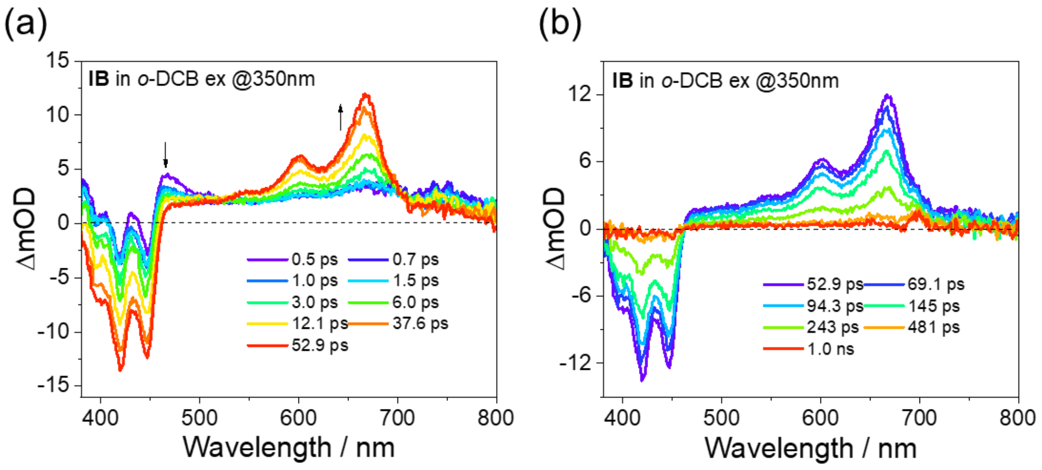

Supplementary Fig. 23 Transient absorption spectra (TAS) under 350 nm excitation. (a) TAS for IB in *o*-DCB under 350 nm excitation between 0.5 ps and 52.9 ps. (b) TAS for IB in *o*-DCB under 350 nm excitation between 52.9 ps and 1.0 ns.

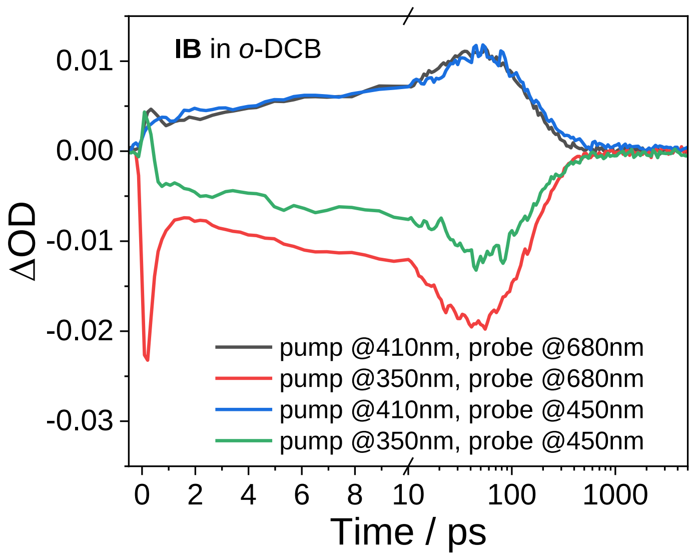

Supplementary Fig. 24 Comparison of the excited-state kinetics. Charge separation kinetics comparison under 410 nm and 350 nm excitation for IB in *o*-DCB.

The TA results of IT-C_{60} in PhCN are shown in **Supplementary Figs. 25** and **26**, which have manifested the accelerated CS and CR processes compared with *o*-DCB.

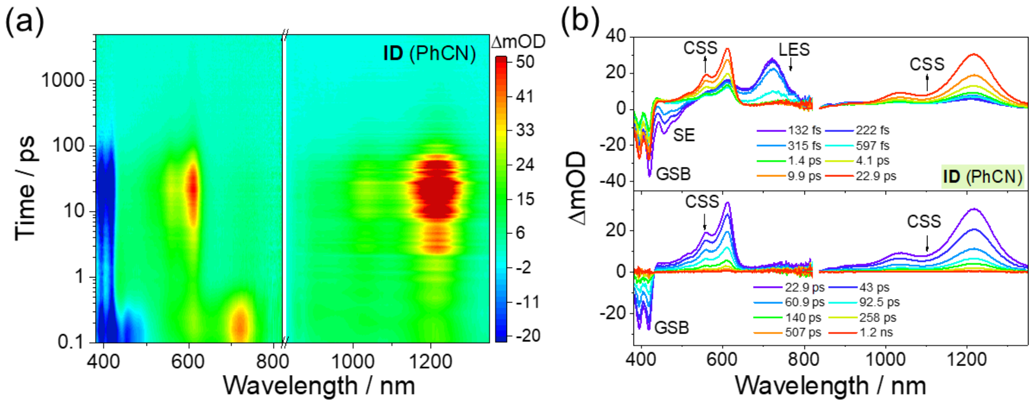

Supplementary Fig. 25 Femtosecond transient absorption results of ID in PhCN. (a) Contour plots following excitation at 410 nm. (b) Selected TA spectra following excitation at 410 nm.

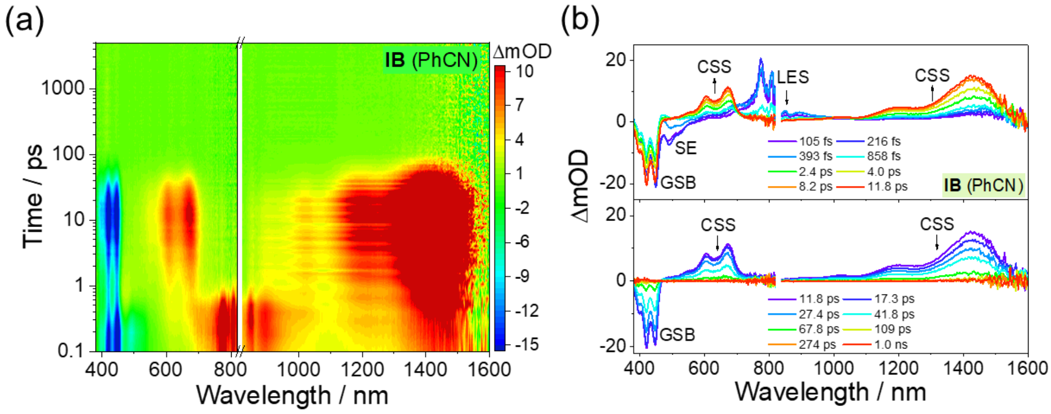

Supplementary Fig. 26 Femtosecond transient absorption results of IB in PhCN. (a) Contour plots following excitation at 410 nm. (b) Selected TA spectra following excitation at 410 nm.

During the charge separation in thin-films, the hole absorption spectra at visible region are normalized within 1 ps, revealing a pronounced spectral narrowing process (**Supplementary Fig. 27**). The hole absorption peak becomes sharper, indicating enhanced ionic character of hole, signifying the evolution from partial charge transfer to charge separation process[12-16]. The kinetics in **Supplementary Fig. 28** suggest that the timescale of spectral narrowing coincides with the completion of charge separation. This implies that charge-separated states (CSS) in the thin-films evolve from partial charge-transfer states (CTS).

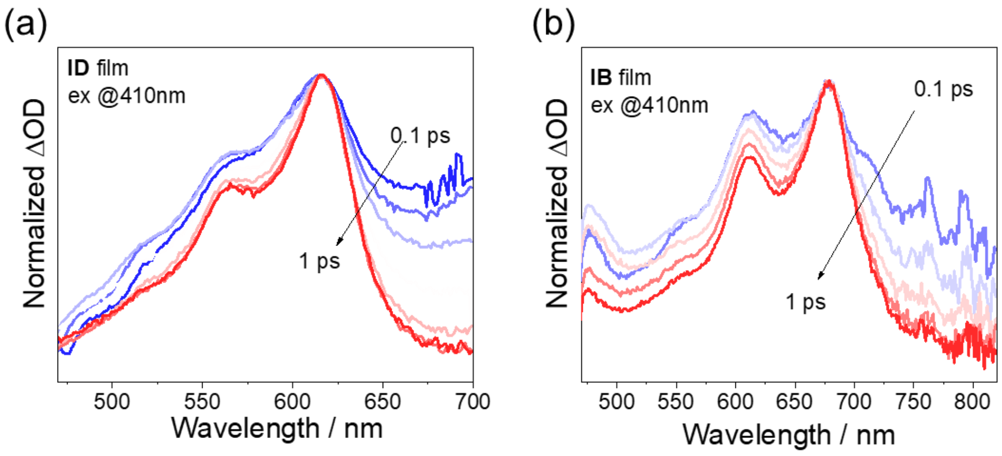

Supplementary Fig. 27 Spectral narrowing process. (a) Spectral narrowing within 1 ps of ID film. (b) Spectral narrowing within 1 ps of IB film.

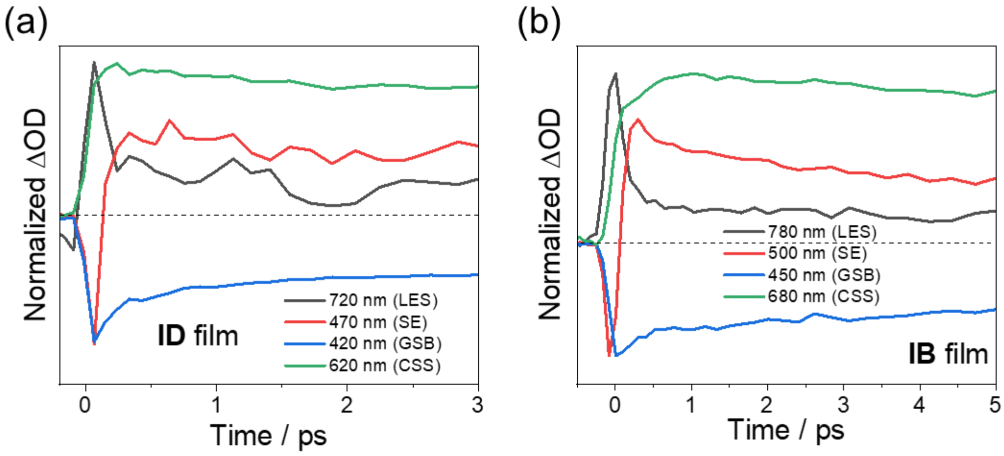

Supplementary Fig. 28 Kinetics of the spectral narrowing process. (a) Kinetics of the hole absorption spectral narrowing process for ID within 3 ps. (b) Kinetics of the hole absorption spectral narrowing process for IB within 5 ps.

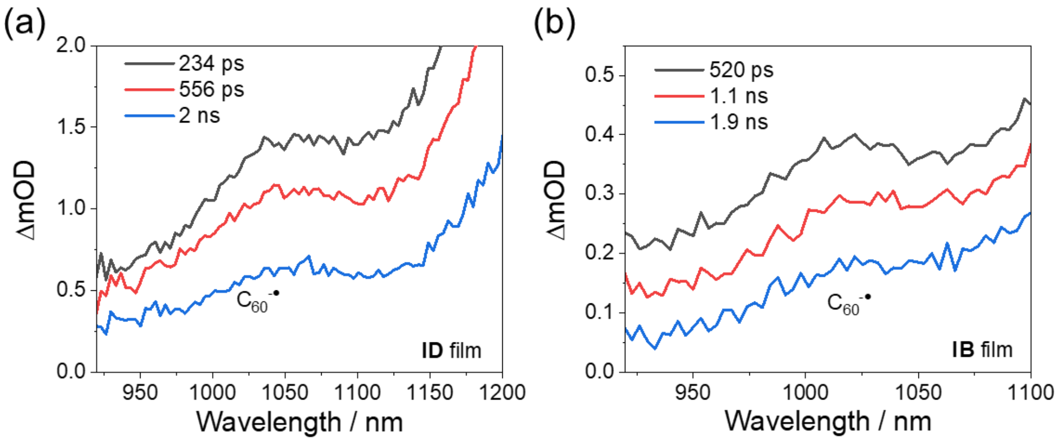

Supplementary Fig. 29 Amplified C_{60} anion ($C_{60}^{-•}$) absorption. $C_{60}^{-•}$ absorption in (a) ID film after 234 ps and in (b) IB film after 520 ps in the NIR region under 410 nm excitation.

Nanosecond transient absorption (ns-TA) of IT-C_{60} in *o*-DCB is shown in **Supplementary Fig. 30**, which has clearly shown that no long-lived CSS exists in *o*-DCB. **Supplementary Fig. 31** presents the comparison between the persistent hole absorption and the $^{3}C_{60}$* absorption in the microsecond timescale, which illustrates that the final state in aggregates is a CSS, rather the localized $^{3}C_{60}$*.

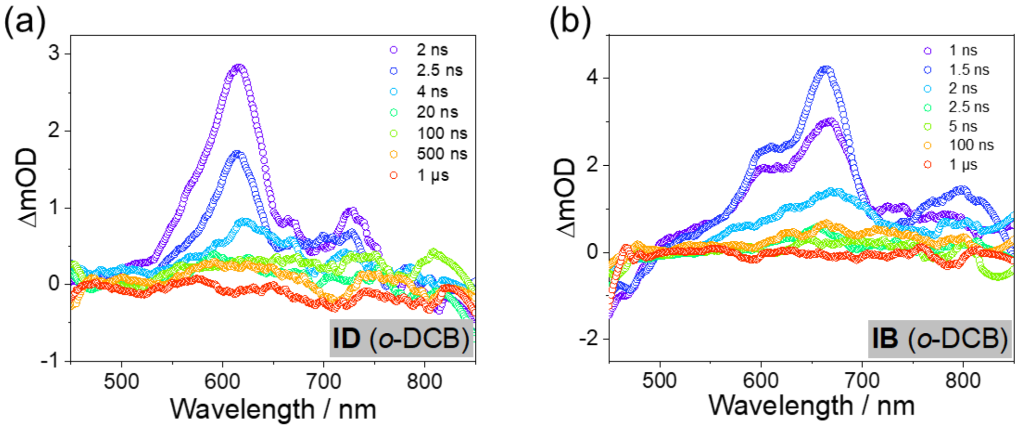

Supplementary Fig. 30 Nanosecond transient absorption spectra in *o*-DCB. (a) Nanosecond transient absorption spectra for ID following excitation at 410 nm. (b) Nanosecond transient absorption spectra for IB following excitation at 410 nm.

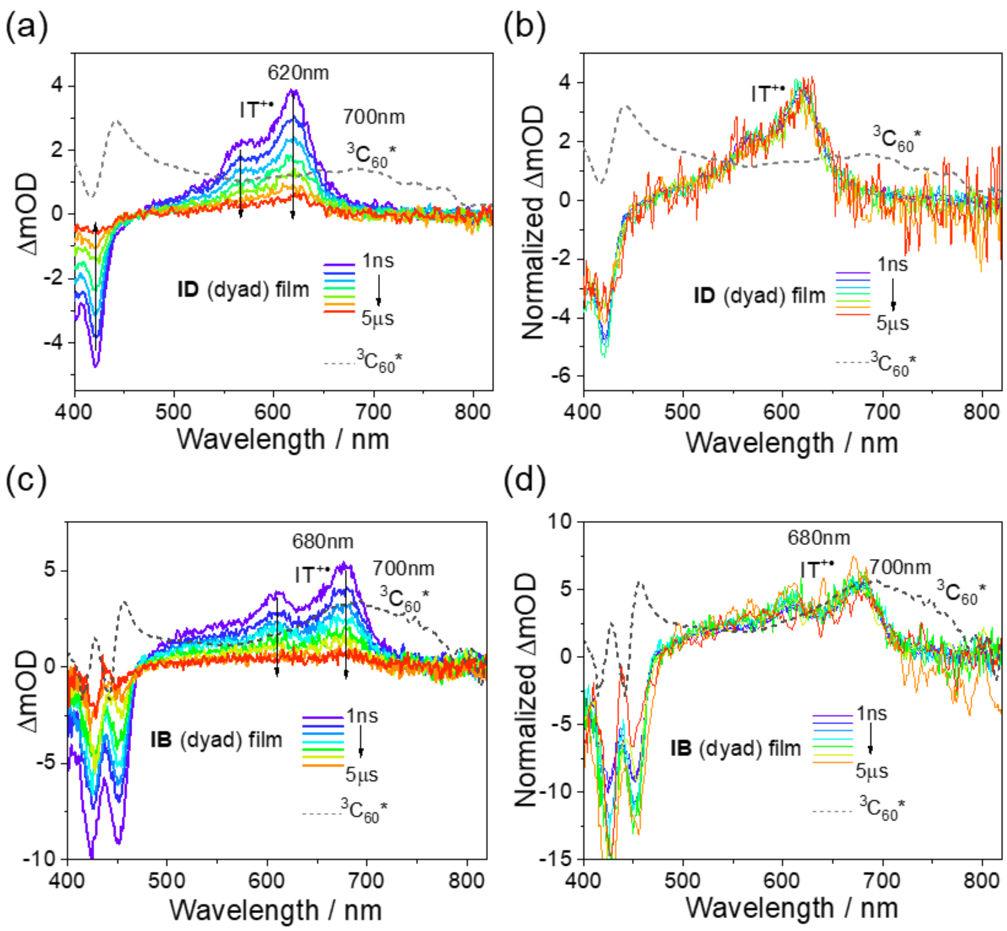

Supplementary Fig. 31 Nanosecond transient absorption spectra in film. (a, b) Nanosecond transient absorption spectra and normalized spectra for ID film following excitation at 410 nm, along with the triplet excited state absorption spectrum of C_{60}. (c, d) Nanosecond transient absorption spectra and normalized spectra for IB film following excitation at 410 nm, along with the triplet excited state absorption spectrum of C_{60}.

The global analysis based on the three-component fitting of transient absorption spectra can be employed in solutions. And the evolution-associated difference spectra (EADS) and corresponding time constants can be obtained. Based on the transient absorption spectra evolution analysis, however, it can be inferred that these are likely not simple sequential processes in the films. Thus, we fit the TA spectra of films for all the probe wavelengths available and obtain the decay-associated difference spectra (DADS) and corresponding time constants[17]. **Fig. 3** and **Supplementary Figs. 32** and

33 presents the DADS (for film) and EADS (for solution) obtained from the original femtosecond and nanosecond transient absorption. Then we show the time-absorption profile at specific wavelength (**Supplementary Figs. 34** and **35**), including the raw kinetic data and fitted results. Fitting kinetic curves are well matched with the raw data at different wavelengths, reflecting the rationality of fitting models. **Supplementary Fig. 36** exhibits the fitted nanosecond transient absorption spectra, which can provide a clearer view of the radical ions absorption features, such as the IDTT cation absorption at 620 and 1250 nm, and C_{60} anion absorption around 1020 nm[18,19].

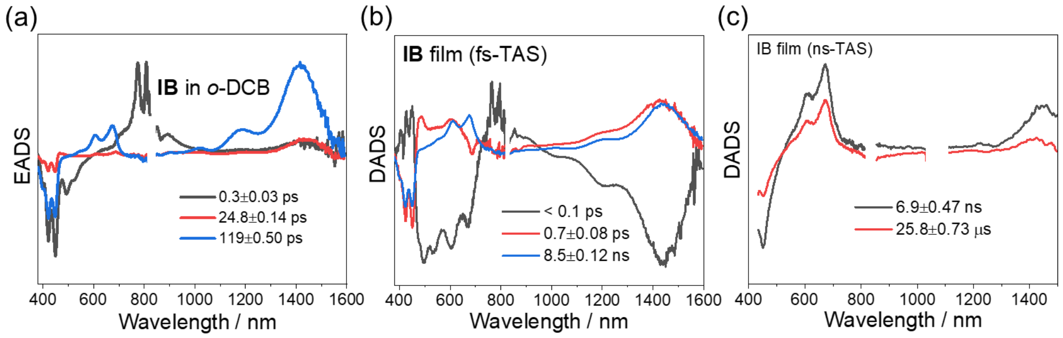

Supplementary Fig. 32 Global and target analysis for IB. (a) Evolution-associated difference spectra (EADS) of IB in *o*-DCB obtained from fs-TA. (b) Decay-associated difference spectra (DADS) of IB film obtained from fs-TA. (c) DADS of IB film obtained from ns-TA.

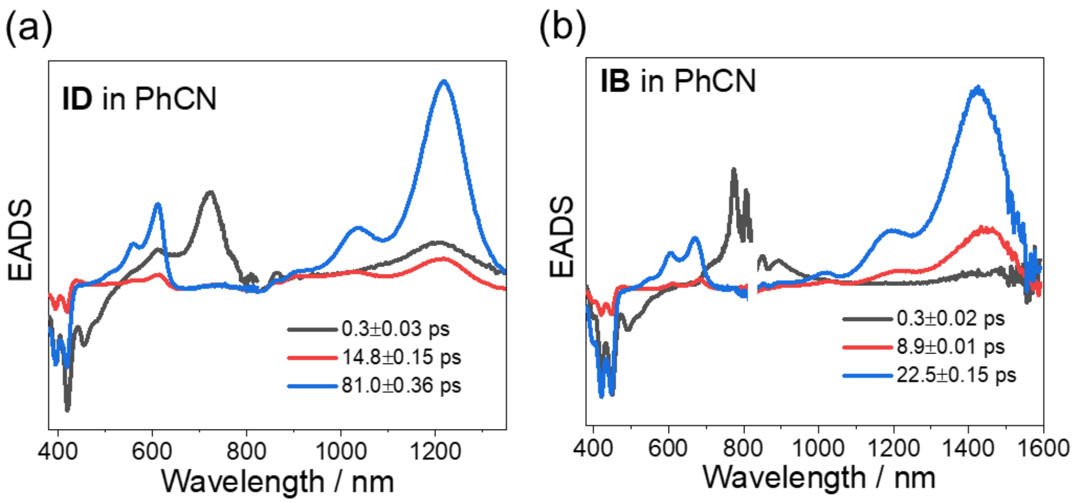

Supplementary Fig. 33 Global and target analysis in PhCN. (a) Evolution-

associated difference spectra (EADS) of ID obtained from fs-TA. (b) Evolution-associated difference spectra (EADS) of IB obtained from fs-TA.

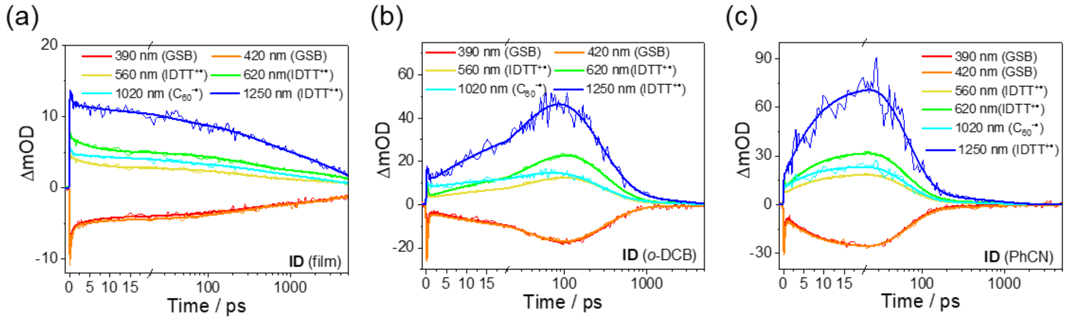

Supplementary Fig. 34 Fitted kinetics for ID. Time-absorption profile at specific wavelength and fitted curves for ID in (a) film, (b) *o*-DCB and (c) PhCN.

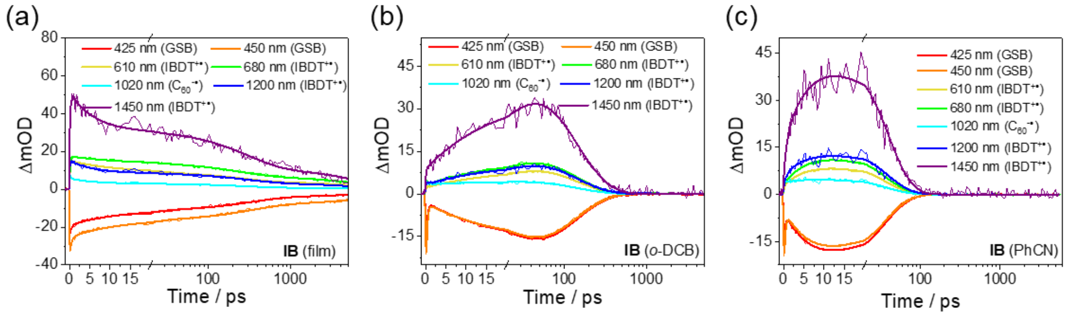

Supplementary Fig. 35 Fitted kinetics for IB. Time-absorption profile at specific wavelength and fitted curves for IB in (a) film, (b) *o*-DCB and (c) PhCN.

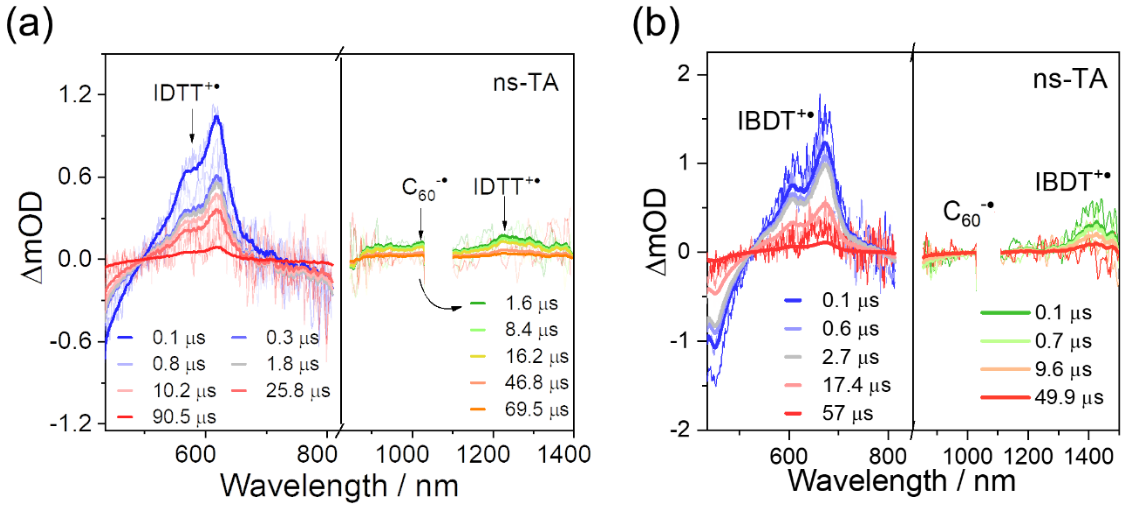

Supplementary Fig. 36 Fitted nanosecond transient absorption spectra. (a) Experimental and fitted nanosecond transient absorption spectra of ID film following 410 nm excitation. (b) Experimental and fitted nanosecond transient absorption spectra of IB film following 410 nm excitation.

The charge separation rate is decreased with the increasing of PMMA doping in the IB film, and resembles the charge separation kinetic of individual molecules in toluene (**Supplementary Fig. 37**). This suggests that intermolecular aggregation accelerates the charge separation of IB, and this is an intermolecular process.

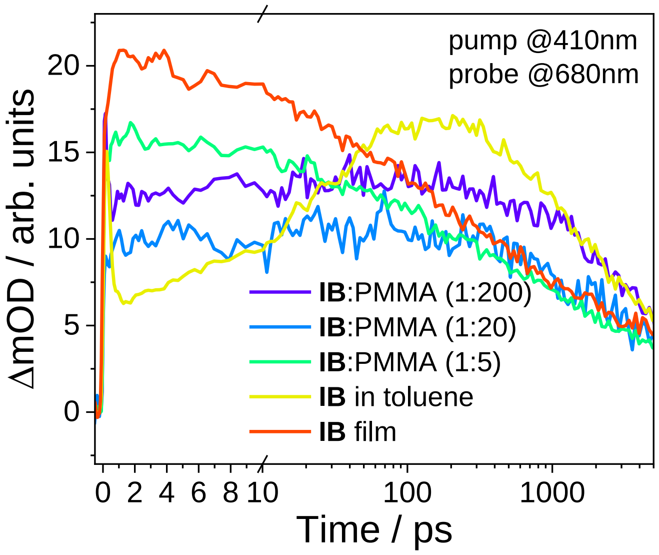

Supplementary Fig. 37 Kinetics analysis of IB in toluene, film and doped with PMMA. Kinetics of CSS in IB film, PMMA-diluted IB film, and IB in toluene within 5 ns timescale, excited at 410 nm and probed at 680 nm.

In thin-films, as the excitation wavelength undergoes a blueshift, i.e., the photon energy increases, charge separation process can be gradually accelerated (**Fig. 4b**, **c** and **Supplementary Fig. 38**). **Supplementary Fig. 39** exhibits the transient absorption spectrum at 0.1 ps after excitation using various wavelengths in IB film. **Supplementary Fig. 40** shows the femtosecond transient absorption spectra of ID and IB thin-films after excited with long-wavelength photon, 520 nm, 570 nm and 650 nm.

Notably, when excited using low-energy photon, the GSB appears upon excitation, following the gradual formation of hole-absorption; but the SE and LES of IT precursors do not generation, and no LES features of C_{60} appears as well, which indicates that low-energy photon excitation mainly populates electrons to CTS, rather than LES[20-22]. This can be further confirmed by the spectral narrowing process (**Fig. 4e-g** and **Supplementary Fig. 41**). However, due to the lack of the hot CTS, dependence of charge separation kinetics on the excitation wavelengths does not exist in solution (**Supplementary Fig. 42**).

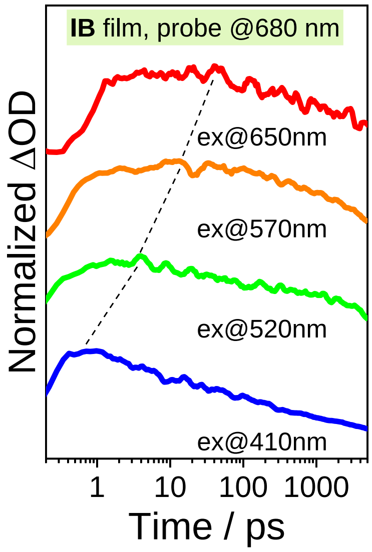

Supplementary Fig. 38 Kinetics analysis under different excitation wavelength. The dependence of charge separation kinetics (probe at 680 nm) on the excitation wavelengths in IB film under 410, 520, 570 and 650 nm excitation.

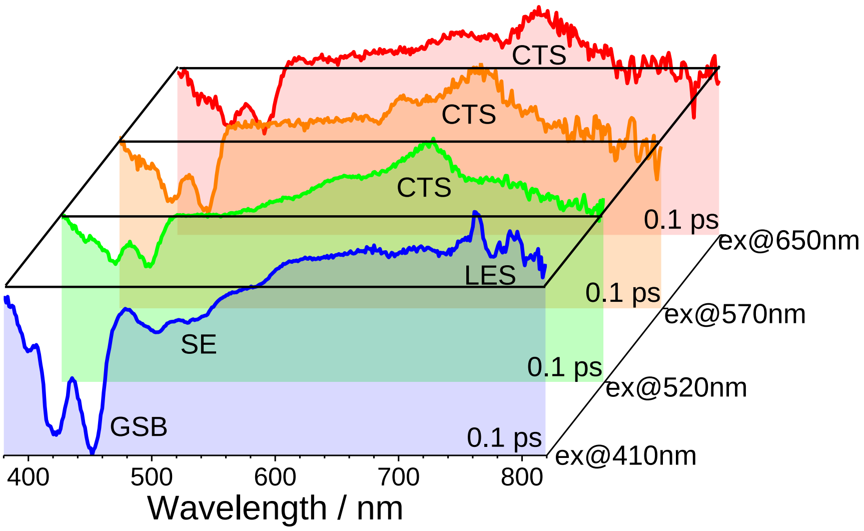

Supplementary Fig. 39 LES and CTS analysis upon various wavelength excitation. Transient absorption spectra within 0.1 ps when the IB film is excited at 410, 520, 570, and 650 nm.

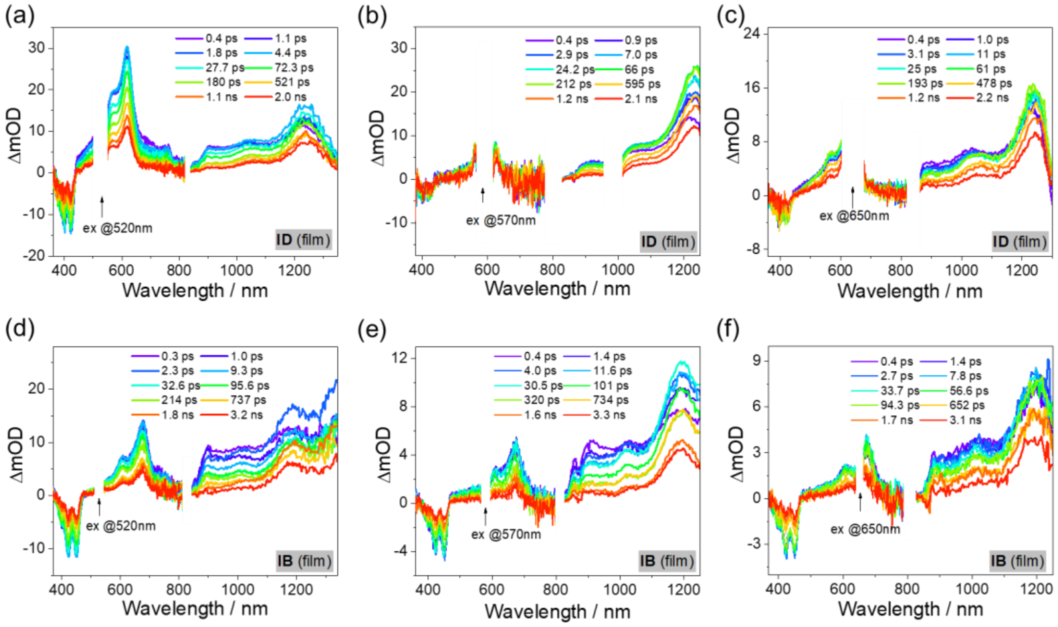

Supplementary Fig. 40 Transient absorption spectra under various wavelength excitation. Femtosecond transient absorption of ID film (a-c), and IB film (d-f) using low-energetic photons excitation: 520 nm, 570 nm and 650 nm.

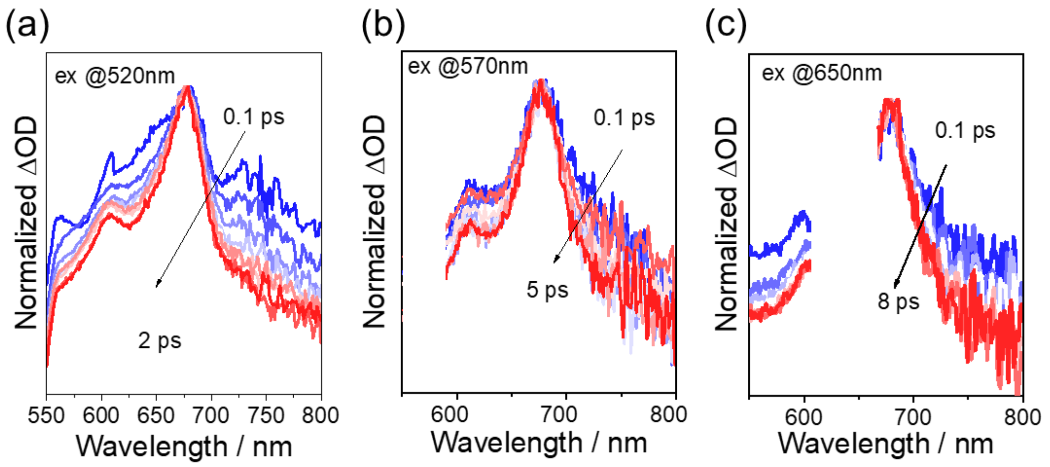

Supplementary Fig. 41 Spectral narrowing analysis. Spectral narrowing analysis process of IB film under (a) 520 nm, (b) 570 nm and (c) 650 nm excitation.

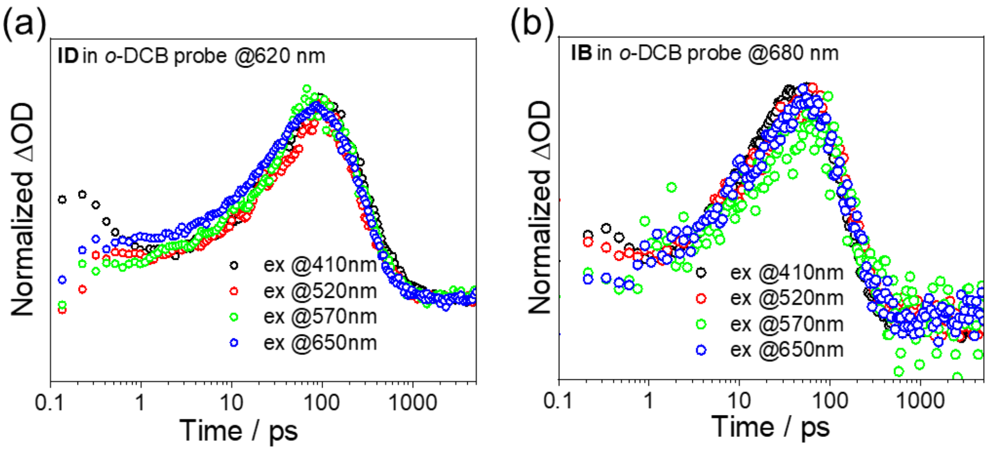

Supplementary Fig. 42 Kinetics under various wavelength excitation in solution. Charge separation kinetics of (a) ID and (b) IB in *o*-DCB solution, under 410 nm, 520 nm, 570 nm and 650 nm wavelength excitations.

Supplementary Fig. 43 shows the dependence of kinetics on excitation density for IB film. Similar to ID, the charge-separated state at nanosecond timescale is intermolecular charge recombination process, while it is intramolecular recombination process at the microsecond timescale.

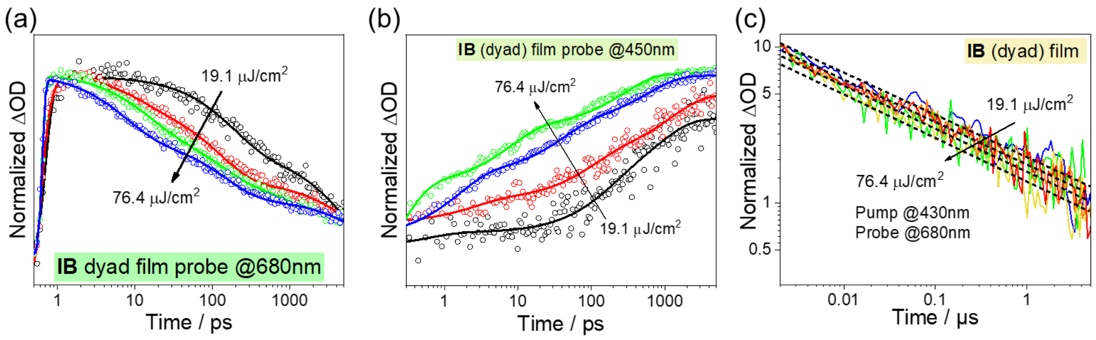

Supplementary Fig. 43 Dependence of kinetics on excitation density in IB film. (a) Charge-separated states (CSS, probe at 680 nm) kinetics following 410 nm excitation under different excitation density within 5 ns timescale. (b) Ground-state bleaching (GSB probe at 450 nm) kinetics following 410 nm excitation under different excitation density within 5 ns timescale. (c) Charge-separated states (CSS, probe at 680 nm) kinetics following 410 nm excitation under different excitation density within 5 μs timescale. The slope of the dashed line reflects the CR rate change.

The dependence of electron transfer kinetics on excitation density for ID and IB in solution are shown in **Supplementary Fig. 44**. Different with aggregates, there is no significant difference of CR kinetics as the excitation density changes, indicating that CR is an intramolecular process in solution[23]. In other words, no carrier intermediate state exists in solution after excited.

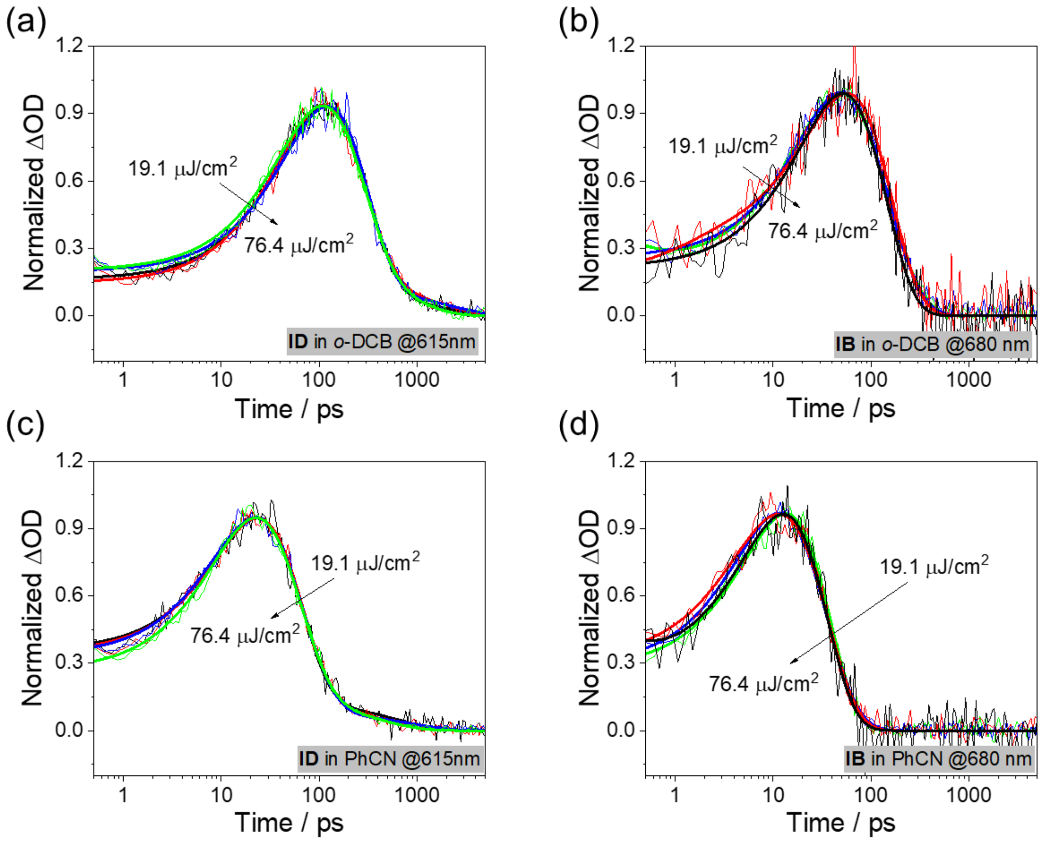

Supplementary Fig. 44 Dependence of kinetics on excitation density in solution. The dependence of electron transfer kinetics (probe at the hole absorption) on excitation density for ID in (a) *o*-DCB and (c) PhCN, and IB in (b) *o*-DCB and (d) PhCN.

The charge separation kinetics of PMMA-diluted ID and IB films (dyad-to-PMMA mass ratio of 1:200) also show no dependence on excitation density, indicating an intramolecular process (**Supplementary Fig. 45**). Consequently, the absence of intermolecular charge separation mediation leads to a notable decrease in the final CSS lifetime with increasing PMMA doping content (**Supplementary Fig. 46**).

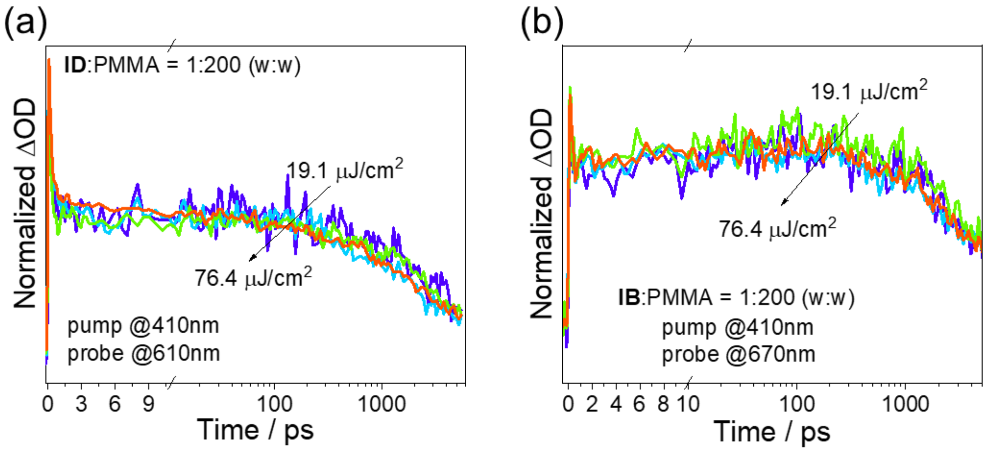

Supplementary Fig. 45 Dependence of kinetics on excitation density after PMMA doping. The dependence of electron transfer kinetics (probe at the hole absorption) on excitation density for (a) PMMA-diluted ID film and (b) PMMA-diluted IB film.

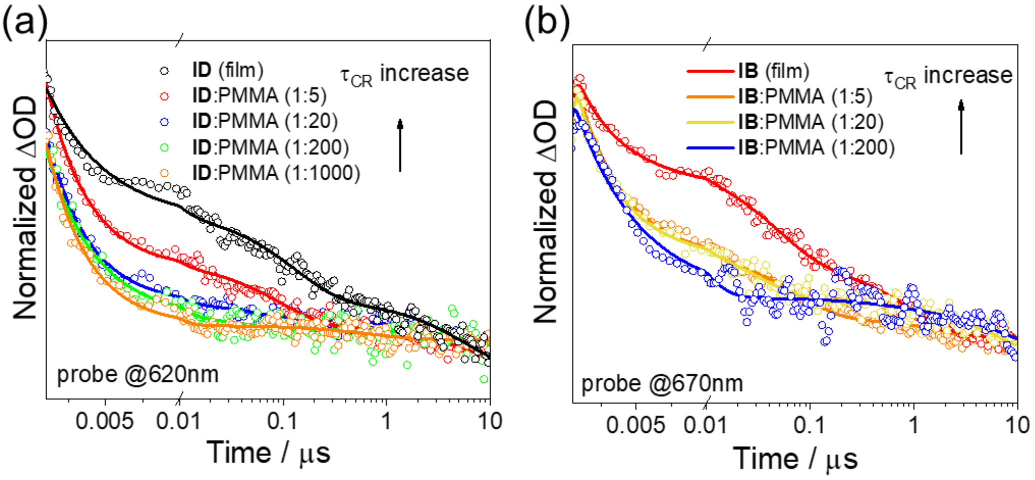

Supplementary Fig. 46 Kinetics under varying PMMA doping. Kinetics of charge-separated state in pure film and PMMA-diluted film for (a) ID and (b) IB within 10 μs timescale.

Furthermore, using the low-energy photon excitation (such as 650 nm), the dependence of charge recombination kinetics on excitation power has obviously decreased (**Supplementary Fig. 47**), compared to high-energy photon excitation (such as 410 nm). This reflects the suppression of free carriers (FCs) generation. As expected, without the efficient FCs mediation, the final charge-separated state lifetime under 650

nm excitation is drastically shorter than that under 410 nm excitation (**Supplementary Fig. 48**).

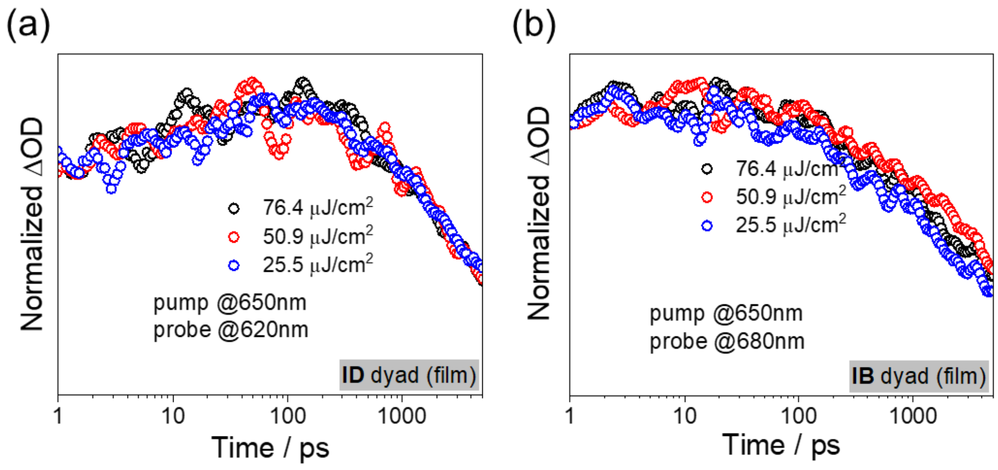

Supplementary Fig. 47 Dependence of kinetics on excitation density following 650 nm excitation. The dependence of electron transfer kinetics (probe at the hole absorption) on excitation power for (a) ID film and (b) IB film under 650 nm excitation.

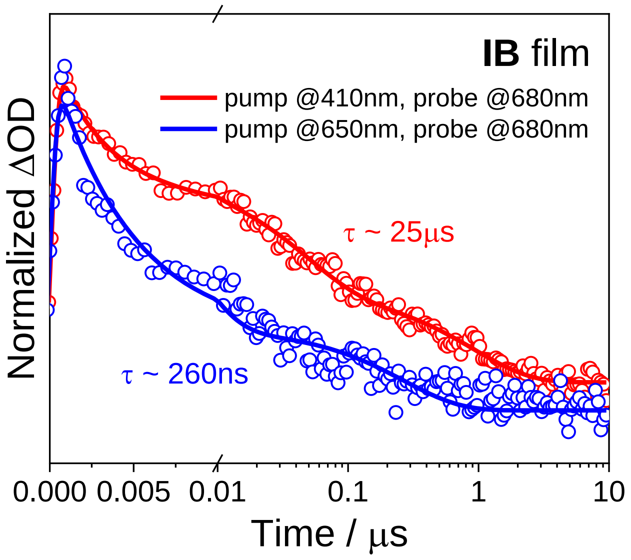

Supplementary Fig. 48 Kinetics upon 410 nm and 650 nm excitation. Kinetics of charge-separated state (probed at 680 nm) within 10 μs timescale for IB dyad film under 410 nm and 650 nm excitation.

The charge-separated state (CSS) kinetics of the IBDT/C_{60} blend aggregates exhibit excitation-density dependence on both nanosecond and microsecond timescales, consistent with the IDTT/C_{60} blend aggregates (**Supplementary Fig. 49**). This illustrates that the final charge recombination in the blends is intermolecular process. Accordingly, the final CSS lifetime in the blend is only 85 ns, compared to the 25 μs in IB dyad (**Supplementary Fig. 50**).

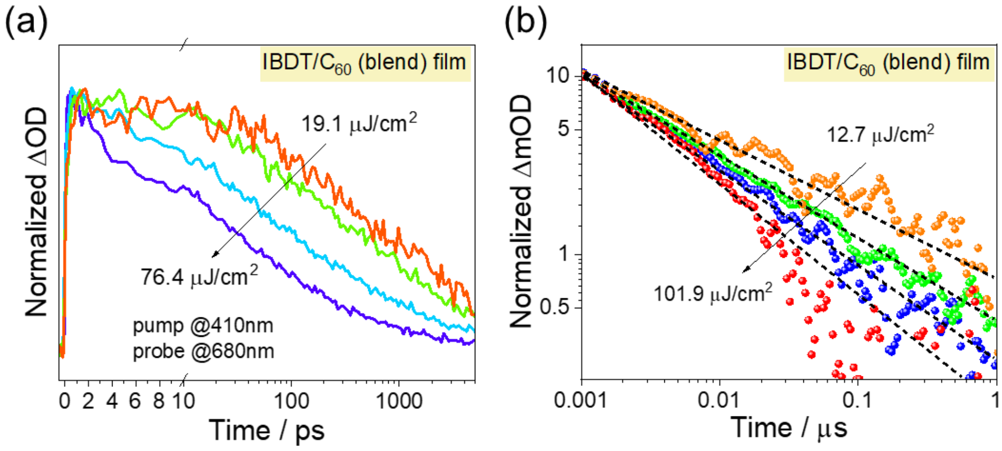

Supplementary Fig. 49 Dependence of kinetics on excitation density for blend films. Dependence of the charge-separated states on excitation density for IBDT/C_{60} blend films within (a) 5 ns and (b) 1 μs timescale excited at 410 nm. The slope of the dashed line in (b) reflects the CR rate change.

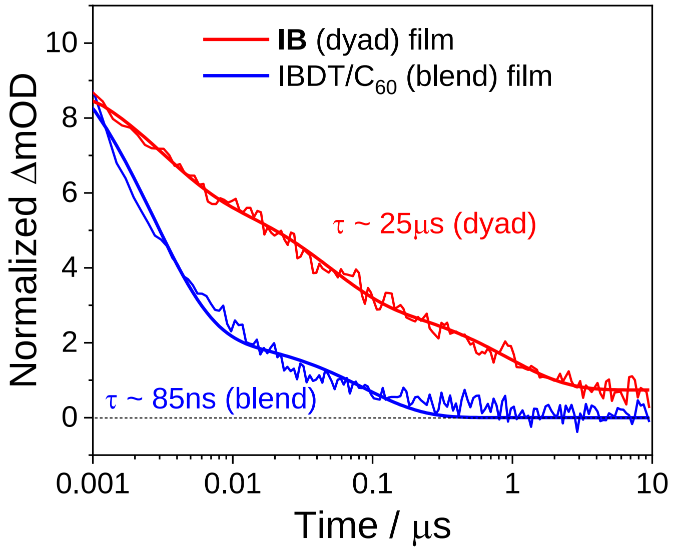

Supplementary Fig. 50 Kinetics comparison between IB dyad and blend film. Comparison of the charge-separated state kinetics between IB dyad and IBDT/C_{60} blend aggregates under 410 nm excitation (19.1 μJ/cm^2).

We investigate the photophysical properties of IT-C_{60} films prepared by "drop-casting, spin-coating, and knife-coating" techniques. As shown in **Supplementary Fig. 51**, it is evident that all films prepared by these techniques exhibit characteristics of fast charge separation and slow recombination, and the kinetic evolution of charge separation states is nearly identical. This, in turn, demonstrates that the ultrafast and long-lived CS processes in small-molecular dyad aggregates are not influenced by the film fabrication procedure, benefitted from the intramolecular charge recombination process of the final charge-separated state.

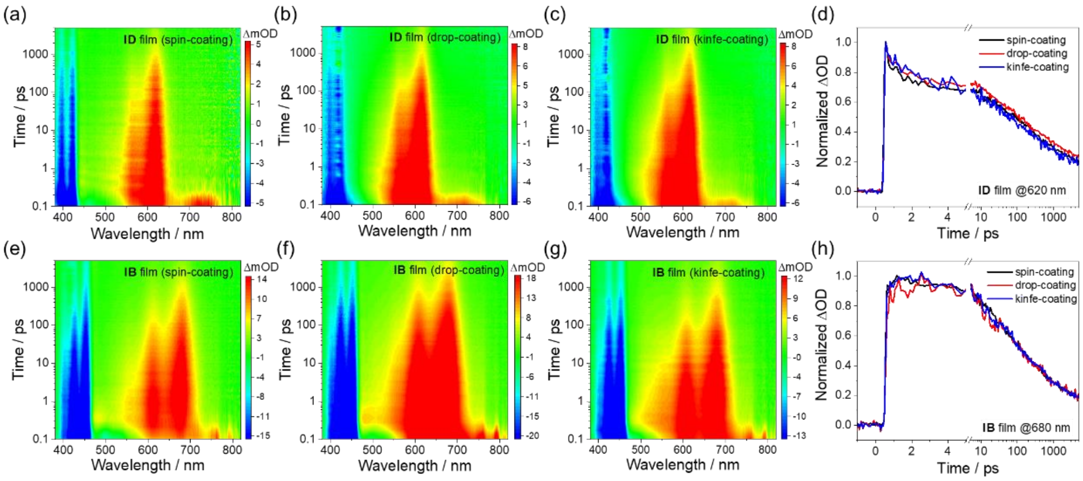

Supplementary Fig. 51 Transient absorption of films under different preparation processes. Transient absorption spectra of (a), (b), (c) for ID film and (e), (f), (g) for IB film under 410 nm excitation, fabricated by spin-coating, drop-coating and knife-coating. Normalized charge separation kinetics of (d) ID and (h) IB film prepared by different fabrication processes.

Supplementary Note 9: Pulsed electron paramagnetic resonance

To further illustrate the ^{3}CSS and its generation mechanism, we employ pulsed electron paramagnetic resonance spectra (pulsed-EPR) measurements taking IB for

example. **Supplementary Fig. 52** shows the contour plot of the pulsed-EPR in IB film under 532 nm excitation at 5 K. Specific discussion and analysis have been described in the text. The pulsed-EPR experiment was conducted under conditions of 5 K and deoxygenation, resulting in a significantly longer CSS lifetime compared to the ns-TA results. Moreover, the pulsed-EPR results can be rationally extended to the results under 410 nm excitation. Because high-energy photon excitation can open up additional hot CTS, which can be resonant with CSS and facilitate FCs generation, thus promoting the intramolecular ^{3}CSS generation mediated by the spin-uncorrelated FCs recombination.

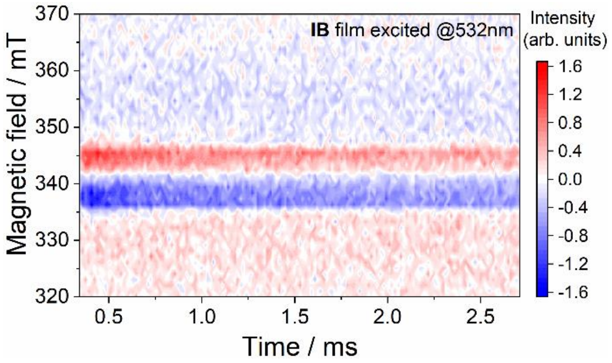

Supplementary Fig. 52 Pulsed-EPR measurement. Contour plot of the pulsed-EPR in IB film under 532 nm excitation at 5 K.

Supplementary Note 10: Computational studies

Supplementary Figs. 53 and **54** shows the HOMO-1, HOMO, LUMO and LUMO+1 distribution of the optimized monomer and dimer for IB and ID, which were calculated at B3LYP-D3BJ/6-31G* level. We do not study the scenario where several monomers aggregation induce long-range charge separation, while it should be pointed out that it can be addressed using the similar method in principle[24]. Using time-dependent DFT at the same level and the Multiwfn program[25,26], the electron-hole distribution of the first 20 singlet excited-states in the monomer and dimer of IB and ID can be exhibited in **Supplementary Figs. 55-58** (isovalue=0.002). Herein, the blue iso-surface represents the electron distribution, and yellow iso-surface represents the hole distribution[21,27]. The charge transfer state (CTS), in which the electrons and holes

are distributed among the acceptor C_{60} and donor IT, respectively, is marked in red, whereas the localized excited state (LES) is marked in blue in **Fig. 6a**. For the LES, there is no obvious difference between electron and hole distribution.

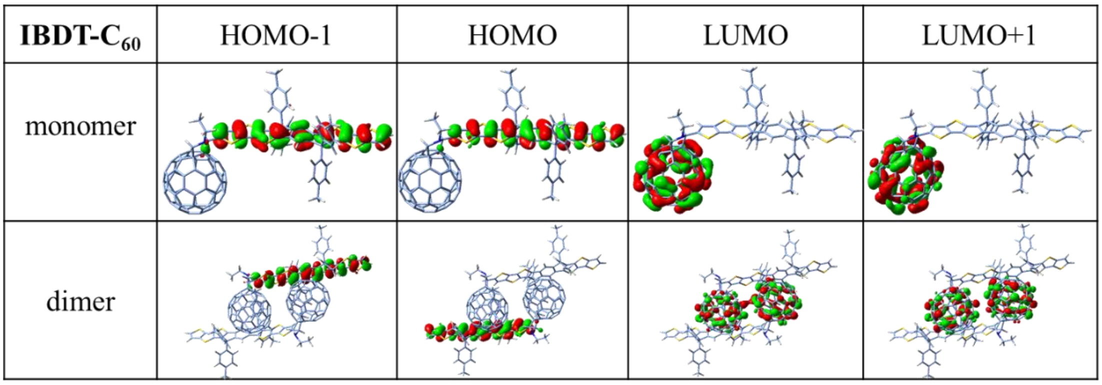

Supplementary Fig. 53 Frontier molecular orbitals distribution. Distribution of the HOMO-1, HOMO, LUMO and LUMO+1 for IB monomers and dimers**.**

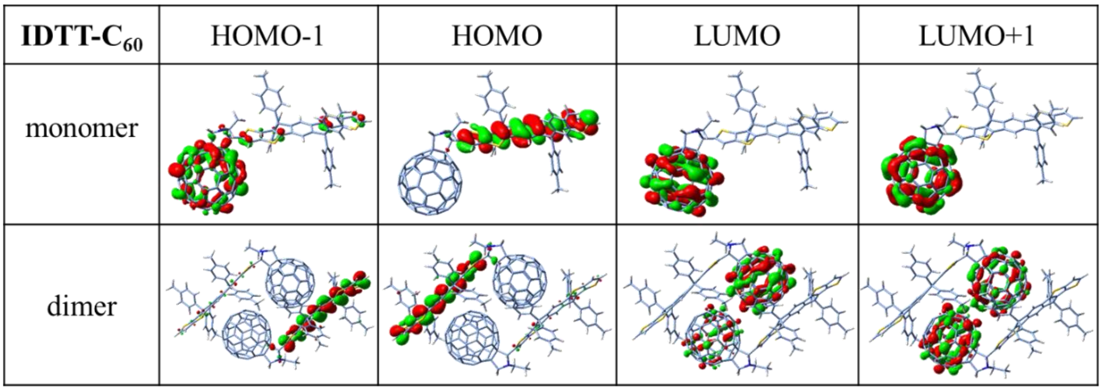

Supplementary Fig. 54 Frontier molecular orbitals distribution. Distribution of the HOMO-1, HOMO, LUMO and LUMO+1 for ID monomers and dimers**.**

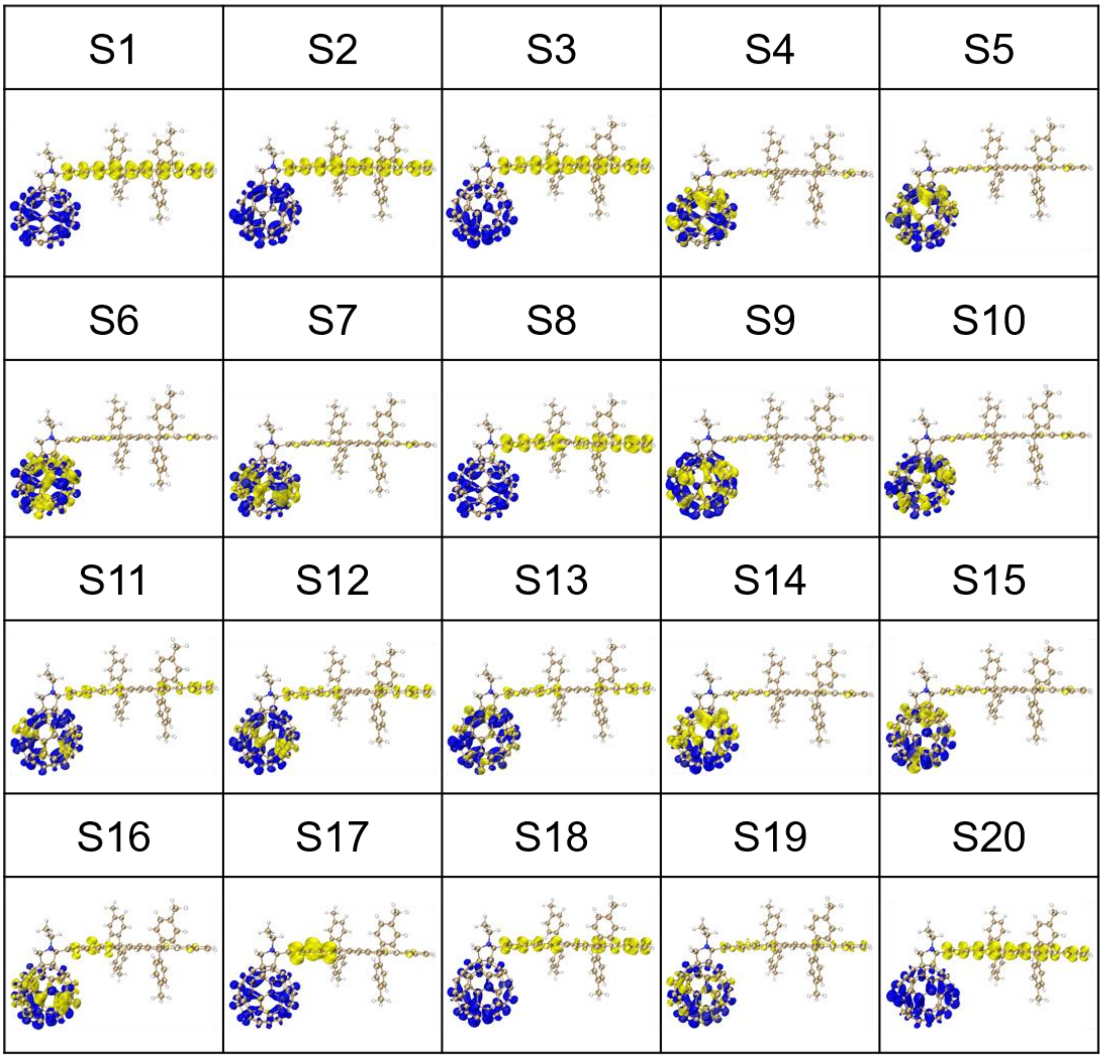

Supplementary Fig. 55 Electron-hole distribution for IB monomer. Electron-hole distribution of the first 20 singlet excited state for IB monomer, in which blue represents electron distribution, and yellow represents hole distribution (isovalue=0.002).

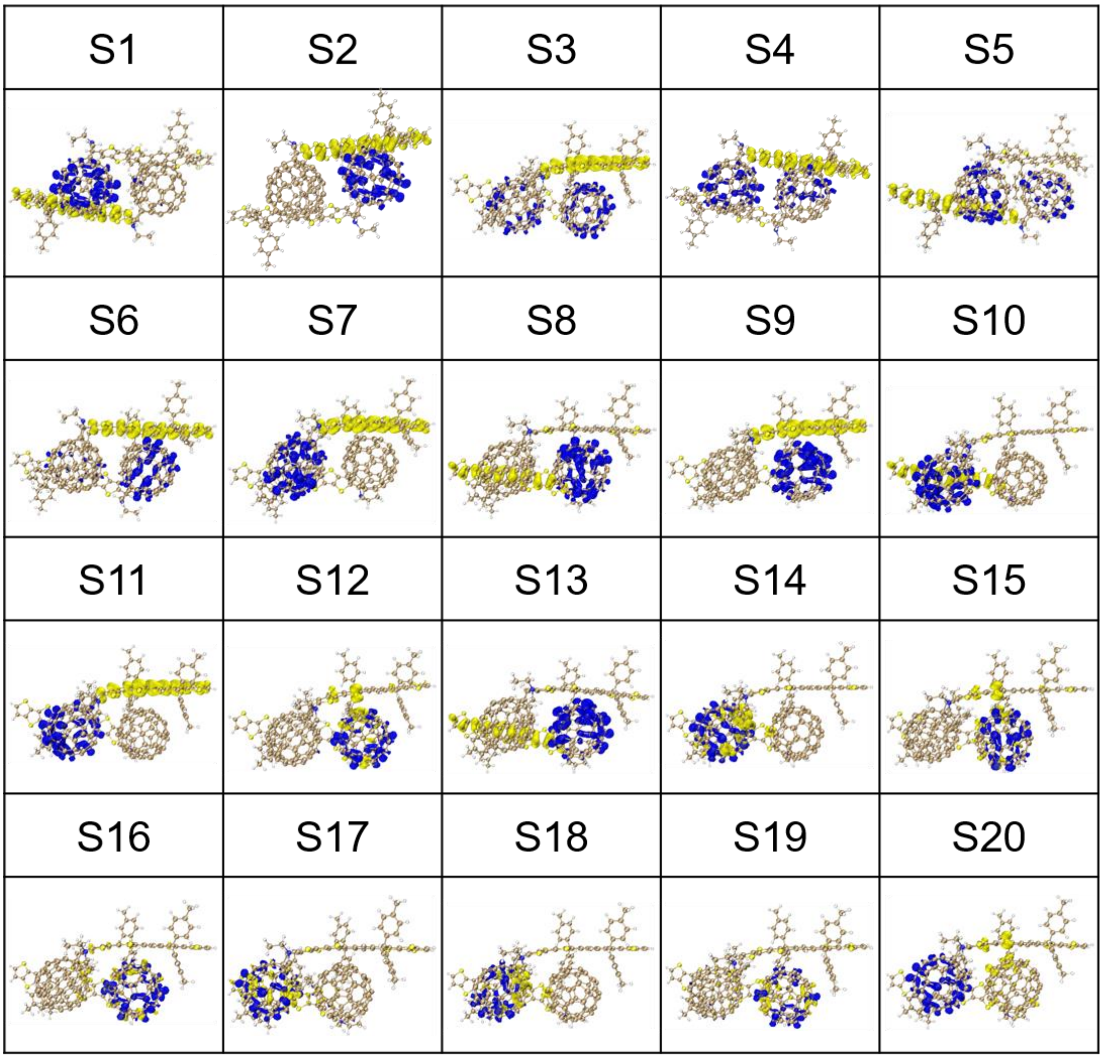

Supplementary Fig. 56 Electron-hole distribution for IB dimer. Electron-hole distribution of the first 20 singlet excited state for IB dimer, in which blue represents electron distribution, and yellow represents hole distribution (isovalue=0.002).

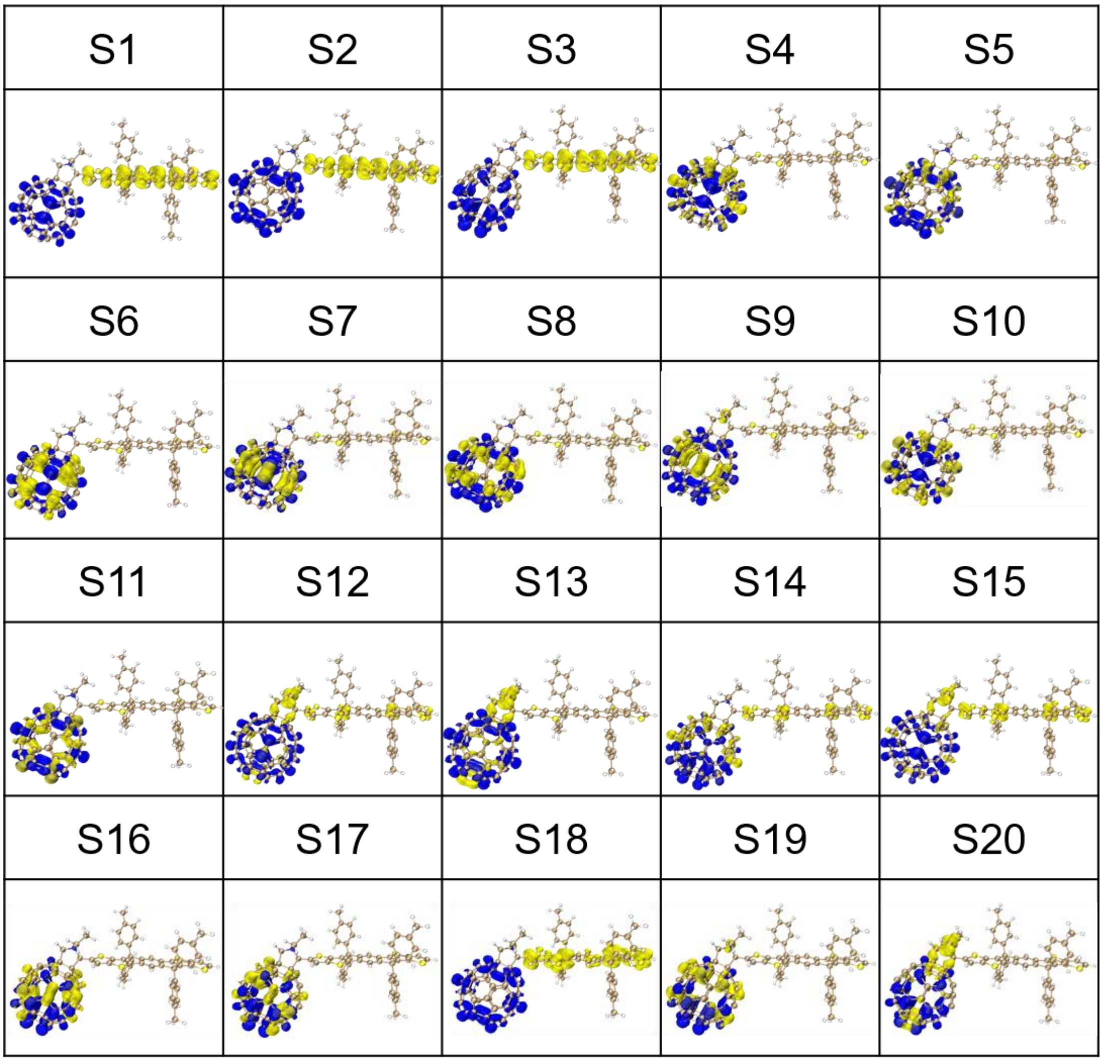

Supplementary Fig. 57 Electron-hole distribution for ID monomer. Electron-hole distribution of the first 20 singlet excited state for ID monomer, in which blue represents electron distribution, and yellow represents hole distribution (isovalue=0.002).

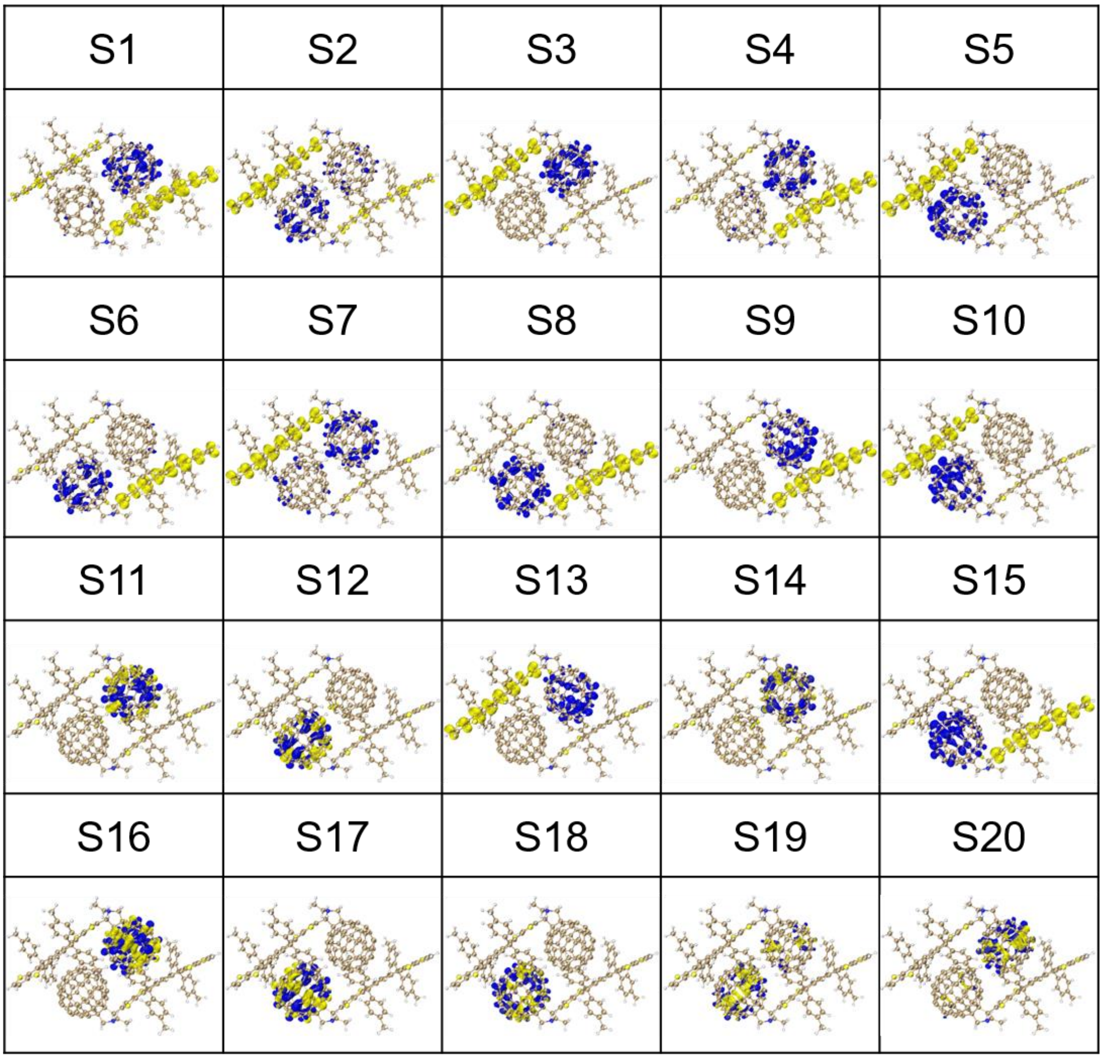

Supplementary Fig. 58 Electron-hole distribution for ID dimer. Electron-hole distribution of the first 20 singlet excited state for ID dimer, in which blue represents electron distribution, and yellow represents hole distribution (isovalue=0.002).

Supplementary Note 11: Photoelectronic switch

Blue-light excitation (410 nm) of the dyad aggregates can open up the additional pathways connecting the intermolecular and intramolecular charge-separated state (CSS), leading to long-lived intramolecular CSS (40 μs for ID dyad and 25 for IB dyad), and achieving ultrafast charge separation (sub-picosecond) at the meanwhile; alternatively, using red-light excitation (650 nm), or in non-aggregated systems (solutions), the intermolecular CS pathway can be closed, resulting in a two-order-of-magnitude decrease in the charge separation rate, and the ultimate CSS lifetime is

shortened from 40 μs to 490 ns (under 650 nm excitation) and 311.4 ps (in solution) for ID dyad. This property may be applicable in the fields such as molecular photoelectronic switches, by modulating the excitation wavelength or aggregation state to open the additional intermolecular CS pathway, thus simultaneously achieving the desired ultrafast CS and long-lived CSS. Schematic diagrams are provided in **Fig. 6**e for ID dyad and **Supplementary Fig. 59** for IB dyad.

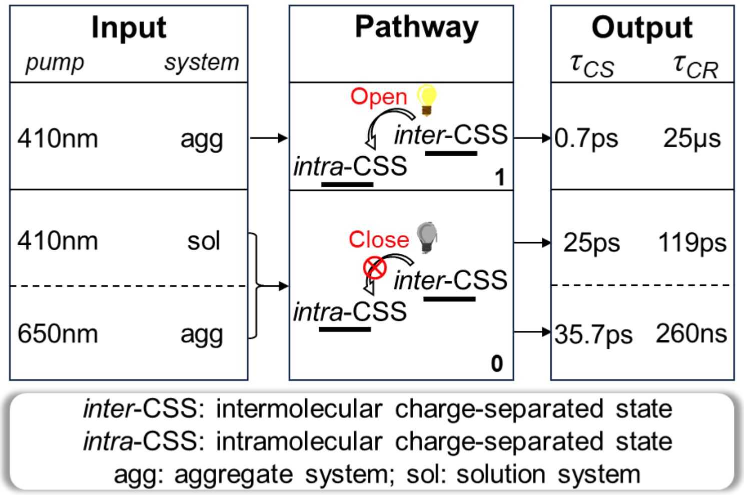

Supplementary Fig. 59 Diagram illustrating the photoelectronic switch of IB dyad film.

Supplementary Note 12: Photocurrent response

Similar to the ID dyad aggregates, photocurrent response of the IB dyad film and the IBDT:C_{60} blend film also indicates a significant increase of the photocurrent density upon the addition of HQ to the electrolyte, but the increase is more dramatic in IB dyads (**Supplementary Fig. 60**). This suggests that IB dyad possesses superior capability in storing transient redox equivalents compared to blends.

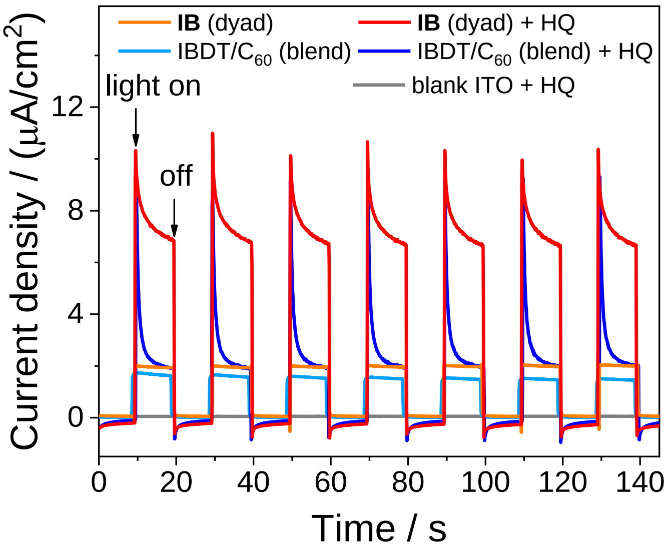

Supplementary Fig. 60 Photocurrent response. Photocurrents for IB dyad film, IBDT:C_{60} blend film and the blank ITO film in 0.2 M Na_2SO_4 with or without 50 mM hydroquinone (HQ) under AM 1.5 illumination (100 mW) at 0 V vs. Ag/AgCl.

Supplementary Reference

1. Maggini, M., Scorrano, G. & Prato, M. Addition of azomethine ylides to C60: synthesis, characterization, and functionalization of fullerene pyrrolidines. *J. Am. Chem. Soc.* **115**, 9798-9799 (1993).
2. Liu, X. *et al.* Unveiling Excitonic Dynamics in High-Efficiency Nonfullerene Organic Solar Cells to Direct Morphological Optimization for Suppressing Charge Recombination. *Adv. Sci.* **6**, 1802103 (2019).
3. Li, Q. *et al.* Insights into Charge Separation and Transport in Ternary Polymer Solar Cells. *ACS Appl. Mater. Interfaces* **11**, 3299-3307 (2019).
4. Yang, C. *et al.* Hot-casting Strategy Empowers High-Boiling Solvent-Processed Organic Solar Cells with Over 18.5% Efficiency. *Adv. Mater*. e2305356, (2023).
5. Guo, Y. *et al.* Bridge-Mediated Charge Separation in Isomeric N-Annulated Perylene Diimide Dimers. *J. Am. Chem. Soc.* **141**, 12789-12796 (2019).
6. Weller, A. Photoinduced Electron Transfer in Solution: Exciplex and Radical Ion Pair Formation Free Enthalpies and their Solvent Dependence. *Z. Phys. Chem.* **133**, 93-98 (1982).
7. Guldi, D. M. & Prato, M. Excited-state properties of C(60) fullerene derivatives. *Acc. Chem. Res.* **33**, 695-703 (2000).
8. Schlenker, C. W. *et al.* Polymer Triplet Energy Levels Need Not Limit Photocurrent Collection in Organic Solar Cells. *J. Am. Chem. Soc.* **134**, 19661-19668 (2012).
9. Kuang, Z. *et al.* Conformational Relaxation and Thermally Activated Delayed Fluorescence in Anthraquinone-Based Intramolecular Charge-Transfer Compound. *J. Phys. Chem. C* **122**, 3727-3737 (2018).
10. Zhang, Z. *et al.* Ultrafast exciton transport at early times in quantum dot solids. *Nat. Mater.* **21**, 533-539 (2022).
11. Holzel, H. *et al.* Probing Charge Management across the pi-Systems of Nanographenes in Regioisomeric Electron Donor-Acceptor Architectures. *J. Am. Chem. Soc.* **144**, 8977-8986 (2022).
12. Kumpulainen, T., Lang, B., Rosspeintner, A. & Vauthey, E. Ultrafast Elementary Photochemical Processes of Organic Molecules in Liquid Solution. *Chem. Rev.* **117**, 10826-10939 (2017).
13. Dereka, B., Koch, M. & Vauthey, E. Looking at Photoinduced Charge Transfer Processes in the IR: Answers to Several Long-Standing Questions. *Acc. Chem. Res.* **50**, 426-434 (2017).
14. Clark-Winters, T. L. & Bragg, A. E. Electron Transfer in Conjugated Polymer Electrolyte Complexes: Impact of Donor–Acceptor Interactions on Microstructure, Charge Separation, and Charge Recombination. *J. Phys.l Chem. C* **126**, 19580-19593 (2022).
15. Sung, J. *et al.* Direct Observation of Excimer-Mediated Intramolecular Electron Transfer in a Cofacially-Stacked Perylene Bisimide Pair. *J. Am. Chem. Soc.* **138**, 9029-9032 (2016).
16. Wu, Y. *et al.* Ultrafast Photoinduced Symmetry-Breaking Charge Separation and Electron Sharing in Perylenediimide Molecular Triangles. *J. Am. Chem. Soc.* **137**,

13236-13239 (2015).
17. Hashimoto, S. *et al.* Ultrafast charge transfer dynamics in the excited state of DCM measured by a 6-fs UV pulse laser. *Chem. Phys.* **551**, 111326 (2021).
18. Rao, A. *et al.* The role of spin in the kinetic control of recombination in organic photovoltaics. *Nature* **500**, 435-439 (2013).
19. Gelinas, S. *et al.* Ultrafast long-range charge separation in organic semiconductor photovoltaic diodes. *Science* **343**, 512-516 (2014).
20. Bakulin, A. A. *et al.* The role of driving energy and delocalized States for charge separation in organic semiconductors. *Science* **335**, 1340-1344 (2012).
21. Grancini, G. *et al.* Hot exciton dissociation in polymer solar cells. *Nat. Mater.* **12**, 29-33 (2013).
22. Tamura, H. & Burghardt, I. Ultrafast charge separation in organic photovoltaics enhanced by charge delocalization and vibronically hot exciton dissociation. *J. Am. Chem. Soc.* **135**, 16364-16367 (2013).
23. Etzold, F. *et al.* The effect of solvent additives on morphology and excited-state dynamics in PCPDTBT:PCBM photovoltaic blends. *J. Am. Chem. Soc.* **134**, 10569-10583 (2012).
24. Fazzi, D., Barbatti, M. & Thiel, W. Unveiling the Role of Hot Charge-Transfer States in Molecular Aggregates via Nonadiabatic Dynamics. *J. Am. Chem. Soc.* **138**, 4502-4511 (2016).
25. Lu, T. & Chen, F. Multiwfn: a multifunctional wavefunction analyzer. *J. Comput. Chem.* **33**, 580-592 (2012).
26. Liu, Z., Lu, T. & Chen, Q. An sp-hybridized all-carboatomic ring, cyclo[18]carbon: Electronic structure, electronic spectrum, and optical nonlinearity. *Carbon* **165**, 461-467 (2020).
27. Liu, L. P. *et al.* Highly efficient photocatalytic hydrogen production via porphyrin-fullerene supramolecular photocatalyst with donor-acceptor structure. *Chem. Eng. J.* **444**, 136621 (2022).
