# Supplementary material for: Aggregation promotes charge separation in fullerene-indacenodithiophene dyad
Source: Nat Commun. 2024 Jul 6;15:5681. doi: 10.1038/s41467-024-50001-z (PMC11227505; doi:10.1038/s41467-024-50001-z)
Supplement: Supplementary file 3 — Description of Additional Supplementary Files [file 41467_2024_50001_MOESM3_ESM.pdf]

Description of Additional Supplementary Files:

Supplementary Data 1: Optimized atomic coordinates for the ID monomer at the B3LYPD3(BJ)/6-31G* level. Structures are given in .xyz file format.

Supplementary Data 2: Optimized atomic coordinates for the ID dimer at the B3LYP-D3(BJ)/6- 31G* level. Structures are given in .xyz file format.

Supplementary Data 3: Optimized atomic coordinates for the IB monomer at the B3LYPD3(BJ)/6-31G* level. Structures are given in .xyz file format.

Supplementary Data 4: Optimized atomic coordinates for the IB dimer at the B3LYP-D3(BJ)/6- 31G* level. Structures are given in .xyz file format.
